# HLA-B27-Homodimer-Specific Antibody Modulates the Expansion of Pro-Inflammatory T-Cells in *HLA-B27* Transgenic Rats

**DOI:** 10.1371/journal.pone.0130811

**Published:** 2015-06-30

**Authors:** Osiris Marroquin Belaunzaran, Sascha Kleber, Stefan Schauer, Martin Hausmann, Flora Nicholls, Maries Van den Broek, Sravan Payeli, Adrian Ciurea, Simon Milling, Frank Stenner, Jackie Shaw, Simon Kollnberger, Paul Bowness, Ulf Petrausch, Christoph Renner

**Affiliations:** 1 Division of Oncology, University Hospital Zurich, Zurich, Switzerland; 2 Functional Genomics Center Zurich, Swiss Federal Institute of Technology Zurich / University of Zurich, Zurich, Switzerland; 3 Division of Gastroenterology and Hepatology, Department of Internal Medicine, University Hospital Zurich, Zurich, Switzerland; 4 Central Biological Laboratory, University of Zurich, Zurich, Switzerland; 5 Department of Rheumatology, University Hospital Zurich, Zurich, Switzerland; 6 Institute of Infection, Immunity and Inflammation, College of Medical, Veterinary and Life Sciences, University of Glasgow, Glasgow, United Kingdom; 7 Department of Oncology, University Hospital Basel, Basel, Switzerland; 8 Nuffield Department of Orthopaedics, Rheumatology and Musculoskeletal Science, University of Oxford, Oxford, United Kingdom; 9 Department of Clinical Immunology, University Hospital Zurich, Zurich, Switzerland; University of East London, UNITED KINGDOM

## Abstract

**Objectives:**

*HLA-B27* is a common genetic risk factor for the development of Spondyloarthritides (SpA). HLA-B27 can misfold to form cell-surface heavy chain homodimers (B27_2_) and induce pro-inflammatory responses that may lead to SpA pathogenesis. The presence of B27_2_ can be detected on leukocytes of *HLA-B27*+ Ankylosing spondylitis (AS) patients and *HLA-B27* transgenic rats. We characterized a novel B27_2_–specific monoclonal antibody to study its therapeutic use in *HLA-B27* associated disorders.

**Methods:**

The monoclonal HD5 antibody was selected from a phage library to target cell-surface B27_2_ homodimers and characterized for affinity, specificity and ligand binding. The immune modulating effect of HD5 was tested in *HLA-B27* transgenic rats. Onset and progression of disease profiles were monitored during therapy. Cell-surface B27_2_ and expansion of pro-inflammatory cells from blood, spleen and draining lymph nodes were assessed by flow cytometry.

**Results:**

HD5 bound B27_2_ with high specificity and affinity (K^d^ = 0.32 nM). HD5 blocked cell-surface interaction of B27_2_ with immune regulatory receptors KIR3DL2, LILRB2 and Pirb. In addition, HD5 modulated the production of TNF from CD4+ T-cells by limiting B27_2_ interactions *in vitro*. In an *HLA-B27* transgenic rat model repetitive dosing of HD5 reduced the expansion of pro-inflammatory CD4+ T-cells, and decreased the levels of soluble TNF and number of cell-surface B27_2_ molecules.

**Conclusion:**

HD5 predominantly inhibits early TNF production and expansion of pro-inflammatory CD4+ T-cells in *HLA-B27* transgenic rats. Monoclonal antibodies targeting cell-surface B27_2_ propose a new concept for the modulation of inflammatory responses in *HLA-B27* related disorders.

## Introduction

Spondyloarthritides (SpAs) are a group of common inter-related inflammatory rheumatic diseases associated with the human leukocyte antigen B27 (*HLA-B27*) allele. Ankylosing spondylitis (AS) is the prototype SpA (~0.5% in the general population) with an 80% *HLA-B27+* prevalence in affected individuals. Other diseases include psoriatic arthritis, enteropathic arthritis in patients with inflammatory bowel disease (IBD), reactive arthritis after specific gastrointestinal and urogenital infections and juvenile SpA [1, 2]. SpA mainly affects the axial skeleton, but peripheral joints and entheses may also be involved. Extra-skeletal manifestations include inflammation of the skin (psoriasis), the gut (IBD) and the eyes (anterior uveitis) [3].

*HLA-B27* is a classical major histocompatibility complex (MHC) class I heterotrimer that presents antigenic peptides to CD8+ T-cells to initiate immune responses. However, unusual biochemical properties of *HLA-B27* include its ability to misfold and form cell-surface β2m-free heavy chain homodimers (B27_2_) [4, 5] that are thought to influence inflammatory responses [6, 7]. It still remains undetermined how the interaction of B27_2_ with immunoregulatory receptors leads to disease. However, given the role that these receptors play in maintaining immune homeostasis, it is suggested that ligation by aberrant forms of B27_2_ may upset this balance in favor of a pro-inflammatory response. Cell-surface B27_2_ has been shown to interact specifically with killer cell Immunoglobulin-like receptors (KIR) and Leukocyte Immunoglobulin-like receptors (LILR) in a manner different from *HLA-B27* heterotrimers [7–9]. In rodents, B27_2_ but not *HLA-B27* binds to related Paired immunoglobulin-like leukocyte receptors (Pir) [10]. Furthermore, it has been demonstrated that interaction of B27_2_ with NK cells and T cells expressing KIR3DL2 results in altered cell signaling, promoting survival and proliferation of Th17 pro-inflammatory cells and protection from apoptosis in NK cells [6, 7]. Cell-surface B27_2_ can be detected in B27-transfected cell lines, AS patients' peripheral blood mononuclear cells (PBMCs) and leukocytes from B27-transgenic rats [7, 10–12].

One of the best-studied animal models for the analysis of *HLA-B27* dependent disease is the ‘33–3’ *HLA-B27* transgenic rat model of colitis [13, 14]. In these rats, pathological progression correlates with the expansion of Th1, Th17 and TNF producing cells [15]. Furthermore, disease development has been shown to be dependent on CD4+ T-cells and gut flora, but independent of CD8+ T-cells [16, 17], suggesting a mechanism of disease that does not depend of *HLA-B27* as an MHC class I molecule presenting antigens to CD8+ T-cells. Additionally, the presence and accumulation of cell-surface B27_2_ in leukocyte populations of HLA-B27 transgenic rats suggests a mechanism that may influence onset and progression of disease [10].

Pro-inflammatory cells expressing tumor necrosis factor (TNF) and interleukin 17 (IL-17) cytokines have been strongly associated with disease development in AS [18–20], and other autoimmune diseases including PsA, RA, IBD and multiple sclerosis (MS) [20–22]. In the clinic, biological treatment of AS relies on TNF inhibitors that have demonstrated to be fairly effective. However, up to 40% of patients do not respond and some patients who initially improve, subsequently lose their response [23–26]. In addition, the efficacy of TNF inhibitors to slow-down structural progression of the disease has revealed conflicting results in different studies [24, 27]. Therefore, there is still an unmet clinical need for additional therapies. Alternative biological treatments targeting various cytokines, cell-surface molecules, and signaling molecules have been assessed [23, 24]. While most biologics have shown little efficacy in SpA, promising early clinical results have been demonstrated with a neutralizing antibody that targets IL-17A in phase II/III development [28], and a monoclonal antibody targeting IL-12/IL-23 in phase II clinical development [29].

We describe the detailed characterization of a novel monoclonal antibody (HD5) that specifically targets cell-surface B27_2_ molecules. Previously, we had described the first B27_2_-specific antibody (HD6) and demonstrated its value to detect B27_2_ molecules *in vitro* [12]. To analysis the impact of B27_2_ recognition and blockade in *HLA-B27* transgenic rats *in vivo*, HD5 antibody was chosen because it binds B27_2_ with higher affinity and specificity.

## Materials and Methods

### Cell lines, antibodies and cytokines

LBL721.220 parental B-lymphocyte-derived cell lines (.220) transfected with B7, B27, B27 C67S and B27 with human tapasin have been described previously [30, 31].

Lymphocyte rat populations were stained with fluorochrome-labelled antibodies or with biotinylated antibodies followed by fluorochrome-labelled streptavidin. Antibodies included were HLA-B27 (HLA-ABC-m3-FITC, AbD serotec, Oxford UK), CD45RA (OX-33-APC-Cy7, Biolegend, San Diego CA), CD3 (G4.18-PE,-biotin, BD-Pharmingen, San Diego, CA), CD3 (G4.18-APC, eBiosciences, San Diego CA), CD4 (OX-35-FITC,-PE-Cy7, BD-Pharmingen), CD8a (OX-8-PerCP, BD-Pharmingen), CD172a (OX-41-FITC, Antibodies-online, Atlanta, GA), anti-granulocytes (RP-1-PE, BD-Pharmingen), CD161a (*10/1-APC, BD-Pharmingen), Mouse IgG1 κ Isotype control (MG1-45, Biolegend), IL-17A (eBio17B7-APC, eBioscience), TNF-α (TN3-19.12-PE, Biolegend), IFN-γ (DB-1-FITC, Biolegend), Brilliant Violet 421-streptavidin (Biolegend). Lineage antibodies included were TCR α/β (R73-Biotin, antibodies-online), Ig (Kpa Chain) (OX-12-Biotin, antibodies-online), CD45RC (OX-22-Biotin, antibodies-online), CD45RA (OX-33-Biotin, Biolegend).

HC10 mAb (IgG2a) was used as a positive control antibody, it binds to β2m-free heavy chains of HLA-B and-C alleles, including both B27_2_ and B27-β2m-free heavy chain forms [31], it was a gift from Dr. Hidde Ploegh (MIT, MA). W6/32 (Abcam, Cambridge UK) recognizes a broad class of β2m-associated HLA-Class I molecules. HD6 mAb (in-house) binds to B27_2_ and free-heavy chains. HD6 and HC10 antibodies were produced by hybridoma cell cultures as previously described [12].

Detection of cell-surface B27_2_ leukocytes by flow cytometry analysis was performed using HD6 mAb amine coupled to a NHS-PEG4-biotin linker (Thermo Scientific, Waltham, MA).

Anti-Her2neu mAb (Trastuzumab, Roche, Switzerland) was used as a control antibody for the *in vivo* study.

Recombinant rat IFN-γ (R&D Systems, Minneapolis, MN) was used at a final concentration of 100 units/mL (32). Recombinant rat tumor necrosis factor α (TNFα) (R&D Systems) was used at a concentration of 3 ng/mL (32). Recombinant rat IL-17A (Abcam) was used at a concentration of 5 ng/mL.

### Production and purification of HD5 mAb

The HD5 Fab antibody was selected from a fully human Fab antibody library (kindly provided by Dyax, MA, USA) as previously described [32]. Fabs were expressed, purified and converted into chimeric IgG1 molecules comprising mouse Fc. A stable transfected NSO cell line carrying the HD5 Ab cassette in a glutamine synthetase gene expression system (LONZA, Switzerland) was generated as previously described [33]. The HD5 (5.1 clone) cell line was grown in DMEM (-/-) supplemented with L-Glutamine (Gibco, Carlsbad, CA), Penicillin-Streptomycin (Gibco), and 10% FBS IgG depleted (PAA, Austria) media. Antibody production was performed in Fibrastage cell culture systems (Eppendorf, Germany). Supernatants were purified using CaptureSelect Fab Kappa matrix (Life technologies, Carlsbad CA). Concentrated IgGs solutions ran through size exclusion chromatography (Hiload 16/60, Superdex 200 PG) (GE Healthcare, UK) in an ÄKTA Prime system (Amersham Pharmacia, UK) and recovered fractions of the purified IgG were desalted, concentrated and sterile filtered.

### HLA complexes

HLA-B*2705 homodimer and heterotrimer complexes were refolded by limiting dilution with or without β2m in the presence of influenza nucleoprotein NP383-391 peptide epitope SRYWAIRTR or EBV EBNA3C epitope RRIYDLIEL as previously described (11). Control HLA heterotrimeric complexes were refolded with the following peptide epitopes: HLA-A*0101 (HLA-A1) with NP 44–52 (CTELKLSDY), HLA-B*0702 (HLA-B7) with hCMVpp65 (TPRTGGGAM), HLA-B*1302 (HLA-B13) with influenza (GILGFVFTL), and HLA-CW*0702 (HLA-CW) with influenza (GILGFVFTL) (11). Streptavidin or Streptavidin-PE (Life technologies) was used to build HLA-tetramers. Biotinylated BSA (Sigma, St. Louis MO) was used in control tetramers and in combinations with HLA complexes.

### ELISA assays

Competition ELISA assays were performed using Maxisorp (Nunc, Switzerland) 96 well plates coated with 2 μg/mL biotinylated BSA, streptavidin (10 μg/mL) (Promega, Switzerland). Biotinylated HLA complexes were incubated for 1 h, blocked with 5% milk powder-PBS, and antibodies were added at 2 μg/mL for 2 h. HRP-conjugated antibodies were used as detectors. Serum detection in ELISA for rat TNF-α ELISA MAX (Biolegend) and IL-17A ELISA MAX (Biolegend) was performed according to manual instructions.

### Surface plasmon resonance analyzes

Surface plasmon resonance (SPR) analyses were performed using Biacore T100 optical biosensor (Biacore, Life Sciences/GE Healthcare, UK). Series S Sensor Chips CM5, Series S Sensor Chips SA, *N*-hydroxysuccinimide (NHS), *N*-ethyl-*N*´-(3-dimethylaminopropyl) carbodiimide (EDC) and 1 M ethanolamine HCl pH 8.5, as well as sampling vials and caps, were obtained from Biacore. Data were collected with the biosensor instrument thermostated to 25°C. A solution of 0.01 M HEPES pH 7.4, 0.15 M NaCl, 0.003 M EDTA, and 0.05% (v/v) Surfactant P20, pH 7.4 (HBS-EP+) was used as running buffer.

#### Protein immobilization

For amine coupling to CM5 chips, a solution of 0.01 M sodium acetate with a pH of one unit below the calculated isoelectric point (pI) of the corresponding protein was used as immobilization buffer. To minimize denaturation, proteins were diluted into the immobilization buffer only immediately before use to final concentrations typically ranging from 10 to 50 μg/mL. Using a flow rate of 5 μL/min, the surface of a flow cell was activated for 7 min using a 1:1 mixture by volume of 0.1 M NHS and 0.4 M EDC. Proteins diluted in immobilization buffer were injected between 20 s and 5 min depending on the desired surface density. Residual activated groups on the surface were blocked by a 7 min injection of 1M ethanolamine-HCl pH 8.5. Proteins were immobilized on SA chips. Proteins diluted in HBS-EP+ to 10 to 50 μg/mL were injected at 10 μL/min for 6 s to 240 s depending on the desired surface density.

#### Kinetic analyses

An initial series of buffer blanks were injected to fully equilibrate the systems. The analyte samples were analyzed from the lowest to the highest concentration and one middle concentration was run in duplicate. To measure the B272 interaction with HD5, 706 response units (RU) of biotinylated B272 were captured on a SA chip surface. During each binding cycle HD5 was injected for 180 s at a flow rate of 30 μL/min and dissociation was monitored for 1.5 h. A concentration range of 0.0625 to 4 nM HD5 was used. To measure the heterotrimer interaction with HD5, 471 RU of HLA-B27 heterotrimers were captured on a SA chip surface. This corresponded to an equimolar amount with respect to the molecular weight ratio between homodimer and heterotrimer. During each binding cycle HD5 was injected for 180 s at a flow rate of 30 μL/min and dissociation was monitored for 0.5 h. A concentration range of 0.0625 to 4 nM HD5 was used. To measure the free heavy-chain interaction with HD5, HLA-B27 free heavy-chain monomers were prepared from the HLA-B27 heterotrimer captured on a SA chip by acidic treatment with 10 mM glycine-HCl pH 2.5. During each binding cycle HD5 was injected for 180 s at a flow rate of 30 μL/min and dissociation was monitored for 0.5 h. A concentration range of 0.0625 to 4 nM HD5 was used.

#### Affinity in solution analyses

To determine the B27_2_ interaction with HD5 by affinity in solution, 26402 RU HD5 were immobilized in a CM5 chip. An initial series of buffer blanks were injected to fully equilibrate the system. This surface was used as a B27_2_ concentration detector. It was calibrated by employing a concentration series of B27_2_ ranging from 0.01 to 1 nM in duplicates. Fixed concentrations of 1 nM B27_2_ were incubated with varying concentrations of 0.01 to 128 nM HD5 for 2h at room temperature in HBS-EP+ in triplicates. Solution equilibrium reaction mixtures were analyzed for the concentration of free B27_2_.

#### HD5 epitope competition

HD5 was coupled to a CM5 chip at a surface density of 26402 RU. HD5, bovine serum albumin (BSA), HD6 and HC10 were injected consecutively at a flow rate of 30 μL/min for 300 s and their binding behavior monitored.

#### HD5 competition with cell-surface receptors

1000 response units (RU) of biotinylated B27_2_ were captured on a SA chip surface. HD5 was injected for 360 s at a flow rate of 30 μL/min and at a concentration range of 6.6 μM. Once the surface was saturated with HD5, surface receptors were injected at concentrations of 4 nM of human KIR3DL2-Fc (R&D systems), 16 nM of human LILRB2 (Sino Biological Inc., China), and 32 nM of mouse Pirb (Sino Biological Inc.) with a flow rate of 30 μL/min, and their binding behavior monitored. During each binding cycle surface receptors were dissociated from B27_2_-chips after injecting 100 mM glycine-HCL for 40 s at a flow rate of 30 μL/min.

#### SPR data processing and analysis

Data sets were processed and analyzed using Biacore T100 Evaluation Software. For kinetic analyses, double referenced association and dissociation phase data were globally fitted to a 1:1 interaction model. As a final processing step, each sensogram was allowed its own bulk refractive index change. For affinity in solution analyses, a plot of the free B27_2_ concentration against the HD5 competitor concentration resulted in the equilibrium dissociation constant.

### Flow cytometry of transfected cells and leukocytes

5 x 10^5^ cells LBL721.220 cells were stained with 0.02 mg/mL of HD5, HD6, HC10, ME1 or W6/32, and for secondary detection 0.02 mg/mL of PE-conjugated goat anti-mouse IgG (Jackson ImmunoResearch, West Grove, PA).

Surface detection of B27_2_ homodimers in leukocytes was performed using HD6-Biotynilated mAb, together with a combination of fluorescent primary antibodies for cell population analysis. Briefly, cells were incubated 1 h in FACS buffer (FB), further Fc Block (anti-CD32, BD Pharmingen) was added for another 1 h at 4 degrees, then 0.1 mg/mL of HD6-Bio for 20 min at RT. Finally, primary antibodies were added for 20 min at 4 degrees followed by 0.01 mg/mL of streptavidin-brilliant violet 405.

Intracellular cytokine staining (ICS) was performed after stimulating leukocytes with Phorbol myristate acetate (PMA) and ionomycin (both at 0.5 μg/mL), in the presence of GolgiPlug (BD bioscience) for the last 5 hours. Cells were fixed in 1% paraformaldehyde, permeabilized with 0.1% saponin in FB, and stained with primary antibodies.

HLA tetramer flow cytometry was conducted using freshly prepared extravidin PE-labeled B27_2_ homodimer tetramers (B27_2_-Tet), HLA-B27 heterotrimer tetramer (B27-Tet) and HLA-B8 heterotrimer tetramer (B8-Tet). To reduce tetramer avidity we constructed HLA-BSA-tetramers were 3 molecules of BSA and 1 molecule of HLA were equimolarly bounded to streptavidin. Tetramers carrying HLA-BSA complexes (B27_2_, B27, B8) are referred as HLA(1x)-tetramers. Tetramers with only BSA were used as controls (BSA-tet). Blocking experiments of leukocytes were assessed with 5 μg of HLA (1x)-tetramers with or without pre-incubation with 0.4 mg/mL of HD5, HC10 and control antibodies (20 min).

Stimulation of leukocytes with tetramers was performed by addition of 0.01 mg/mL HLA-tet or HLA-BSA-tet into cell cultures for 2 days, followed by addition of GolgiPlug overnight.

Flow cytometry analysis was performed using a flow cytometer canto II (BD Bioscience) and data was analyzed using Flowjo version 7.6.4.

Co-culture experiments were performed using LBL721.220 cells and naïve CD4+ T-cells from WT or Tg rats. A total of 100,000 gamma-irradiated 220 APCs were incubated with CD4+ T-cells (1:1 ratio).

*In vitro* cytokine exposure assays were performed with the addition of cytokines for 2 days followed by flow cytometry analysis of cell-surface B27_2_ in different leukocyte populations.

### Animals and *in vivo* experimental design

*HLA-B27*/hβ2m transgenic (33–3 line) (Tg) [13], and wild-type (WT) Fischer F344 male rats were obtained from Taconic (Germantown, NY). Animal experiments were performed according to Swiss federal and cantonal laws on animal protection. Study procedures were approved by the Institutional Cantonal Animal Care and Use Committees (IACUC); cantonal veterinary office Zurich No. 184/2012. Animals were housed under OHA (optimal hygiene area) with *ad libitum* access to food and water.

Rats divided into three groups were randomly assigned to treatment with HD5 mAb or anti-Her2neu accordingly: a. WT-littermates (n = 8), b. Tg-HD5 (n = 10), c. Tg-ctrl (anti-Her2neu) control group (n = 10). Each Tg rat received a weekly intraperitoneal (i.p.) injection of HD5 or anti-Her2neu (ctrl) (10 mg/kg of body weight) up to the age of 15 weeks or 23 weeks. WT-littermates received no treatment (n = 8). Each *HLA-B27* transgenic rat was weighted and monitored every second day for clinical manifestations of colitis (diarrhea), arthritis (red and swollen hind paws), nail dystrophy and psoriasis. The assessment of these individual parameters for each animal was recorded using numerical scores: stool character observations (normal stool = 1, soft stool = 2 and diarrhea = 3); clinical signs of arthritis (normal = 0, mild = 1, moderate = 2, severe = 3) scores for hind paw swelling (0 to 3) and for erythema (0 to 3). Psoriasis was also scored from 0–3, where 0 = without, 1 = present and scattered, 2 = moderate, medium size patches scattered, 3 = severe [34]. Data obtained from individual rats in the different experimental groups were summarized by calculating the group mean and standard deviation (mean ± SD).

0.5 mL of blood was collected every three weeks before the start of treatment and from then on till the end of the mAb therapy for ICS analysis and plasma collection. Bleeding was performed via puncture of the Vena sublingualis and under isoflurane anesthesia.

Rats that show behavioral or physical abnormalities such as paralysis, stress, anxiety, listlessness, signs of infection, abdominal breathing, hunched back, ruffled fur or bodyweight loss of more than 20% were euthanized [35].

Animals were euthanized by CO_2_ inhalation in designed CO_2_ chambers for rats.

### Collection of tissues and histological scores

Paraffin-embedded sections from formalin fixed tissues were sectioned (5 μm), and stained with hematoxylin and eosin. Rat tissues were scored individually by an independent, experienced investigator blinded to the type of treatment. The total histological score represents the sum of the epithelial and infiltration score and thus ranges from 0 to 8 (total score). Histology was scored as follows: Epithelium = 0: normal morphology; 1: loss of goblet cells; 2: loss of goblet cells in large areas; 3: loss of crypts; 4: loss of crypts in large areas. Infiltration = 0: no infiltrate; 1: infiltrate around crypt basis; 2: infiltrate reaching to lamina muscularis mucosae; 3: extensive infiltration reaching the lamina muscularis mucosae and thickening of the mucosa with abundant oedema; 4: infiltration of the lamina submucosa. Statistical analyses for histology were performed using Sigma Plot. Bonferroni-corrected Mann-Whitney rank sum test was applied for animal experiments.

### Statistics

Differences between 2 groups were analyzed by using paired t-test. In experiments with more than two conditions, one-way analysis of variance (ANOVA) for repeated measurements was used, followed by Bonferroni post-hoc multiple comparison test. Significance of results was accepted at p≤0.05. Data are expressed as means ± S.E.M.

## Results

### HD5 mAb specifically binds to B27_2_ homodimers

**Fig 1:**
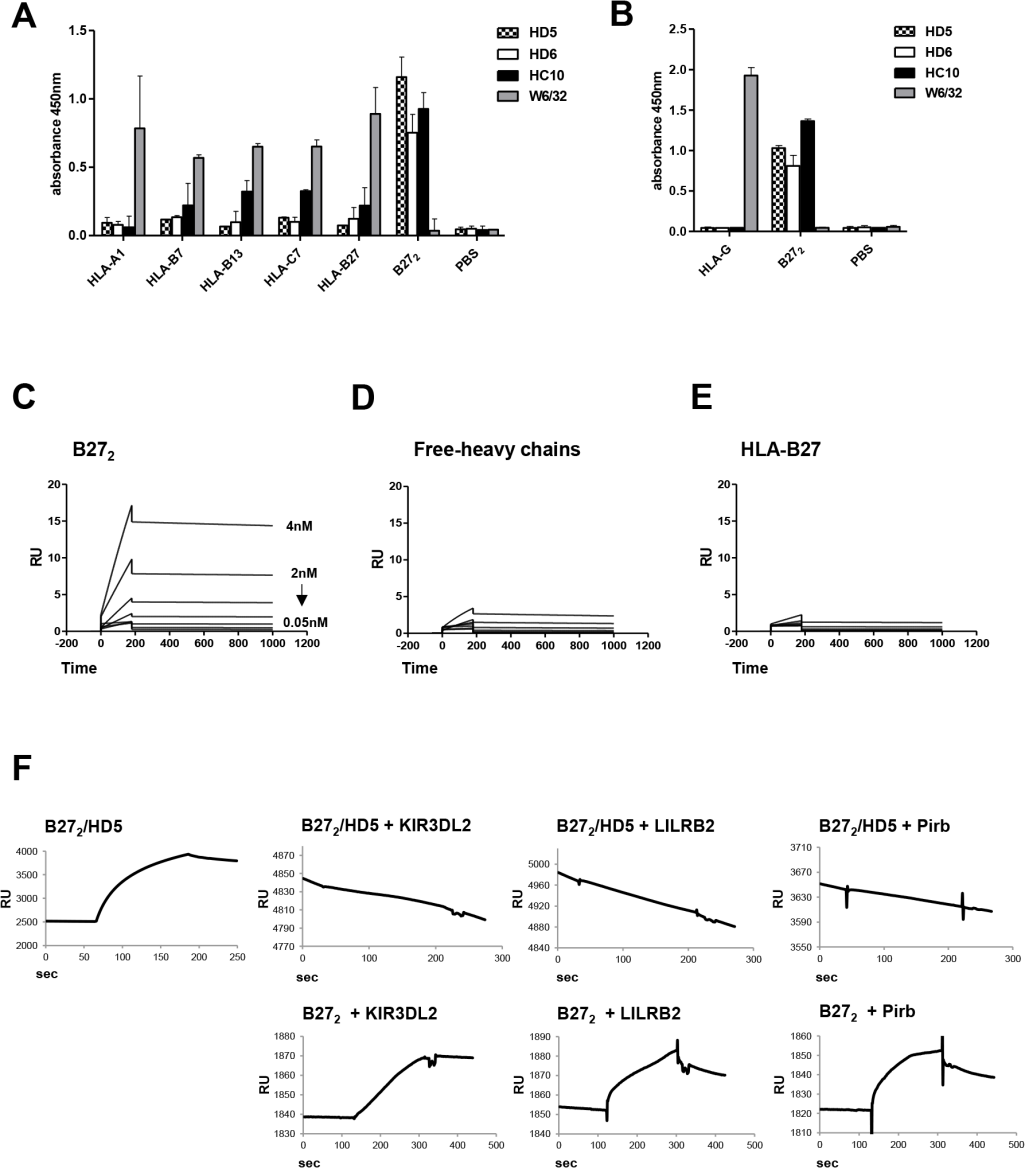
HD5 mAb is specific to B27_2_, binds to recombinant B27_2_, and blocks interaction with immunoregulatory cell receptors. **(A)** ELISA results showed the specificity of HD5 and HD6 to B27_**2**_ homodimers when challenged for different recombinant HLA class I complexes (-A1,-B7,-B13,-C7,-B27, and-B27_**2**_). Control HC10 and W6/32 antibodies were used as positive and negative control, respectively. **(B)** Direct ELISA against HLA-G and B27_**2**_ homodimers using HD5, HD6, HC10 and W6/32 antibodies showed specificity of HD5 and HD6 to immobilized B27_**2**_ homodimers but not to HLA-G homodimers. **(C-E)** Recombinant B27_**2**_, B27-free-heavy chain and HLA-B27 heterotrimers were immobilized into chips for kinetic characterization by SPR. **C)** HD5 and ligand (B27_**2**_) have a K^d^ of 0.32 nM. Immobilized free-heavy chains **(D)** or HLA–B27 heterotrimers **(E)** did not interact with HD5 and K^d^ values were not fitted. **(F)** Blocking competition experiments in SPR were performed by immobilizing B27_**2**_ to a streptavidin chip followed by injection of HD5 to form B27_**2**_-HD5 complexes. Injections of LILRB2, KIR3DL2 and Pirb were assessed and binding events recorded.

We performed an ELISA assay with plate-bound HLA-complexes (Fig 1A) to determine the binding specificity of HD5 and HD6, and compared them with existing antibodies recognizing β2m-free-heavy chains (HC10) and HLA-Class I molecules (W6/32). HD5 and HD6 were shown to be specific for recombinant B27_2_ (Fig 1A); the positive control antibody HC10 bound to B27_2_, while negative control W6/32 did not. We next analyzed by ELISA the binding of HD5 and HD6 to the non-classical MHC molecule HLA-G which also forms homodimers [36] to demonstrate that both antibodies did not cross-react to other HLA dimeric forms and only bound to B27_2_ (Fig 1B). Epitope competition experiments using surface plasmon resonance (SPR) revealed that HD5 bound to a unique B27_2_ epitope that is different from HD6 and HC10, since no competition was observed between antibodies (S1 Fig).

### Kinetics of HD5 binding measured by Surface Plasmon Resonance (SPR)

After determining the specificity for B27_2_, we next assessed the affinity of HD5. Three recombinant variants of HLA-B27 complexes (B27_2_, B27-free-heavy chains and HLA-B27) were immobilized on sensor chips and kinetic analyses were performed (Fig 1C–1E). HD5 interactions with B27_2_ resulted in equilibrium dissociation constant (K^d^) of 0.32 nM, and no interaction with B27-free-heavy chains or HLA-B27 was determined.

To compensate for avidity effects of the IgG format and to determine the monomeric binding constants, we performed in solution competition equilibrium tests using antibody (HD5 and HD6) and ligand (B27_2_) by SPR. HD5 resulted to a K^d^ of 0.159 nM (S2 Fig).

### HD5 inhibits the interaction of cell-surface receptors to B27_2_

We next determined if HD5 was capable of inhibiting the interaction of known immune regulatory receptors binding to B27_2_ by SPR. Competition experiments were performed by saturating B27_2_ chips with HD5 to show that formation of B27_2_/HD5 complexes inhibited the interaction of huKIR3DL2, huLILRB2 and msPirb receptors to B27_2_ (Fig 1F), thus demonstrating the capacity of HD5 as a blocking antibody.

### Cell-surface binding of HD5 to B27_2_ homodimers

**Fig 2:**
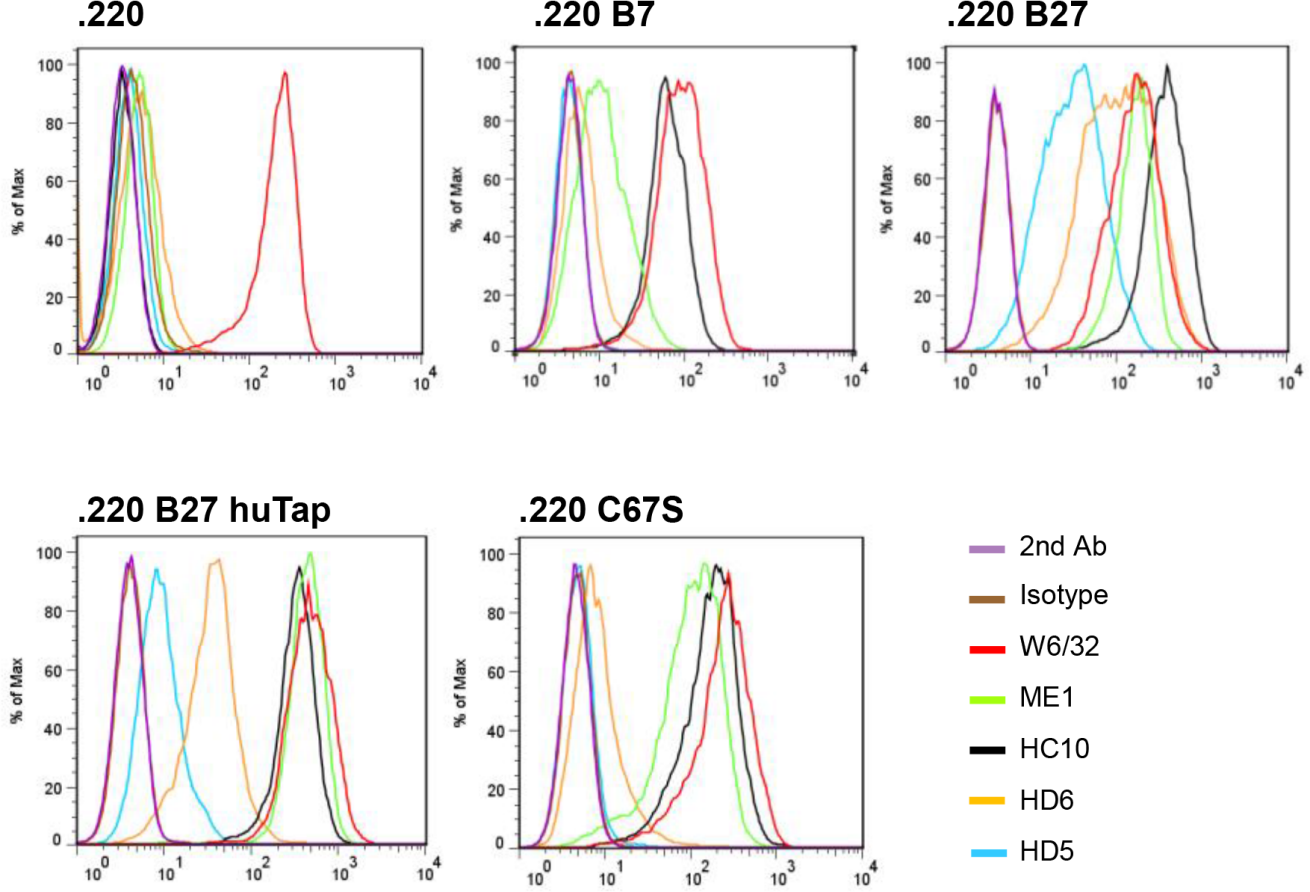
HD5 binds specifically to cell-surface B27_2_ homodimers expressed in human .220 B cell lines. Representative flow cytometry analysis of HD5 and control antibodies (IgG1 Isotype, W6/32, ME1, HC10, and HD6) binding to LBL721.220 cells either untransfected (.220), or transfected with *HLA–B7*, *B27*.*C67S* (*HLA-B27* with a mutation of Cys-67 to serine that abrogates cell-surface B27_**2**_ expression), *B27*.*hutpn* (*HLA-B27* together with transfection of human tapasin, that reduces B27_**2**_ expression and increases HLA-B27 heterotrimer formation), or *HLA-B27* (*HLA-B27* transfected cells expressing high levels of cell-surface B27_**2**_). RU = responsive units. ELISA values are expressed as mean±SEM.

We next asked if HD5 interacted with cell-surface B27 β2m-free heavy chain forms. For that purpose, we used the human LBL721.220 cell line (.220) [11, 37], transfected with *HLA-B27* (.220 B27 cells) that leads to high expression levels of B27 β2m-free heavy chain forms (B27_2_ and B27-free heavy chains) [4]. HD5 bound strongly to .220 B27 cells (Fig 2) but not to control cells. Binding was greatly reduced in the presence of human tapasin, which reduces expression of B27 β2m-free heavy chain forms and increases the expression of HLA-B27 heterotrimers [8]. Furthermore, HD5 did not bind to .220 C67S cells (lacking the free cysteine present in B27 heavy chains) suggesting that HD5 specifically recognizes the B27_2_ conformation form that is dependent on cysteine 67 disulphide linkage [4]. ME1 and W6/32 bound to all cell lines indicating the presence of HLA heterotrimers, while HC10 bound to cells that present HLA-β2m-free heavy chains.

### HD5 blocks the interaction of cell-surface receptors to B27_2_ and modulates TNF expression in CD4+ T-cells *in vitro*

**Fig 3:**
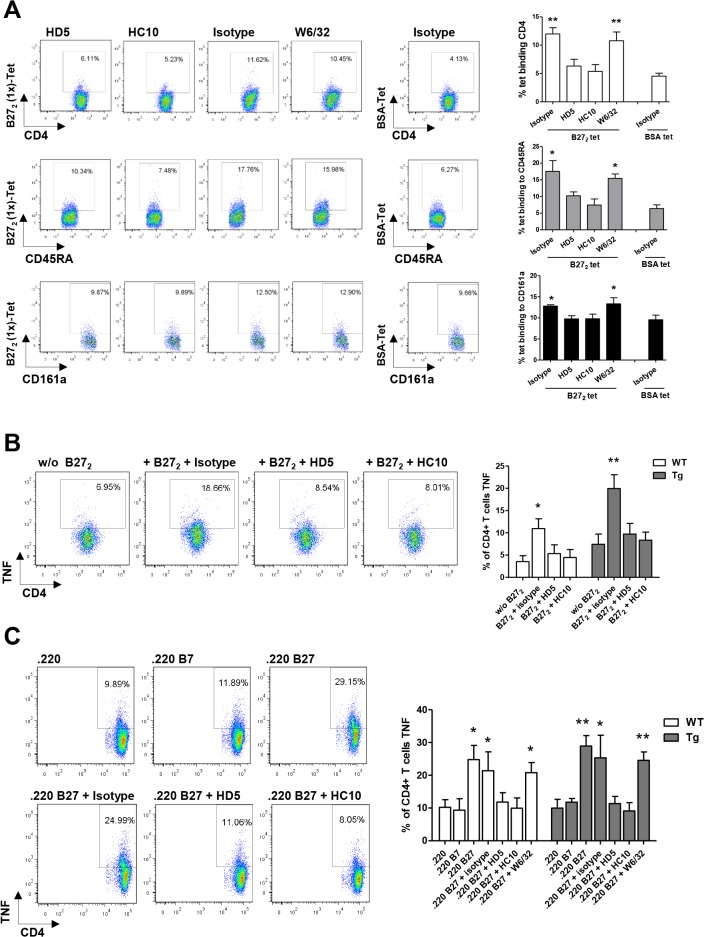
HD5 mAb blocks interaction of B27_2_ to leukocytes and modulates activation of CD4+ T-cells by B27_2_. **(A)** B27_**2**_ (1x)-tetramers were shown to bind to CD4+ T-cells, B-cells (CD45RA,-lineage) and NK (CD161a,-lineage) populations. Pre-incubation of HD5 or HC10 with B27_**2**_(1x)-tetramers partially blocked interactions with cells when compared to control antibodies (W6/32 and isotype). **(B)** Tg and WT CD4+ T-cells produce TNF after incubation with B27_**2**_ (1x)-tetramers. Pre-incubation of HD5 or HC10 with B27_**2**_ (1x)-tetramers inhibited the interaction to CD4+ T-cells and the production of TNF. **(C)** Tg and WT CD4+ T-cells produce TNF after incubation with .220 B27cells expressing B27_**2**_ dimers, but not with .220 and .220 B7 cells. Pre-incubation of HD5 or HC10 with .220 B27 cells blocked the interaction of B27_**2**_ to CD4+ T-cells and the production of TNF. Representative flow cytometry plots belong to leukocytes populations of Tg rats. Tests were performed in triplicates. Tet = tetramer. Values are expressed as mean±SEM. *p<0.05, **p<0.01, as determined by one-way ANOVA followed by Bonferroni post-hoc analysis.

We analyzed the binding of B27_2_ (1x)-tetramers to rat splenocytes with or without pre-incubation of antibodies to further characterize the blocking capacity of HD5. B27_2_ (1x)-tetramers were shown to bind to CD4+ T-cells, B and NK cell populations (Fig 3A) and this interaction was partially blocked when B27_2_ (1x)-tetramers were pre-incubated with HD5 or HC10 (positive control) (Fig 3A).

To rationalize the use of HD5 in a B27 transgenic pre-clinical model, we established an *in vitro* functional assay. Results showed that binding of B27_2_ (1x)-tetramers to CD4+ T-cells from both Tg and WT rats stimulated cells to express TNF (Fig 3B). Furthermore, addition of B27_2_ (1x)-tetramers incubated with HD5 demonstrated that B27_2_/HD5 complexes reduced the production of TNF from CD4+ T-cells (Fig 3B). B27_2_ (1x)-tetramers interaction to rat CD4+ T-cells failed to stimulate significantly the production of IL-17 and IFN-γ (S3 Fig).

Additionally, co-culture of naive CD4+ T-cells from WT or Tg rats with .220, .220 B7 and .220 B27 cells [6, 7] demonstrated that the expression of B27_2_ in .220 B27 cells induced the production of TNF from CD4+ T-cells (Fig 3C). Furthermore, TNF production was blocked when .220 B27 cells were pre-incubated with HD5 (Fig 3C). .220 B27 cells did not induce significantly the production of IL-17 or IFN-γ from rat CD4+ T-cells (S4 Fig).

### B27_2_ expression increases with age in leukocyte populations of *HLA-B27* transgenic rats

**Fig 4:**
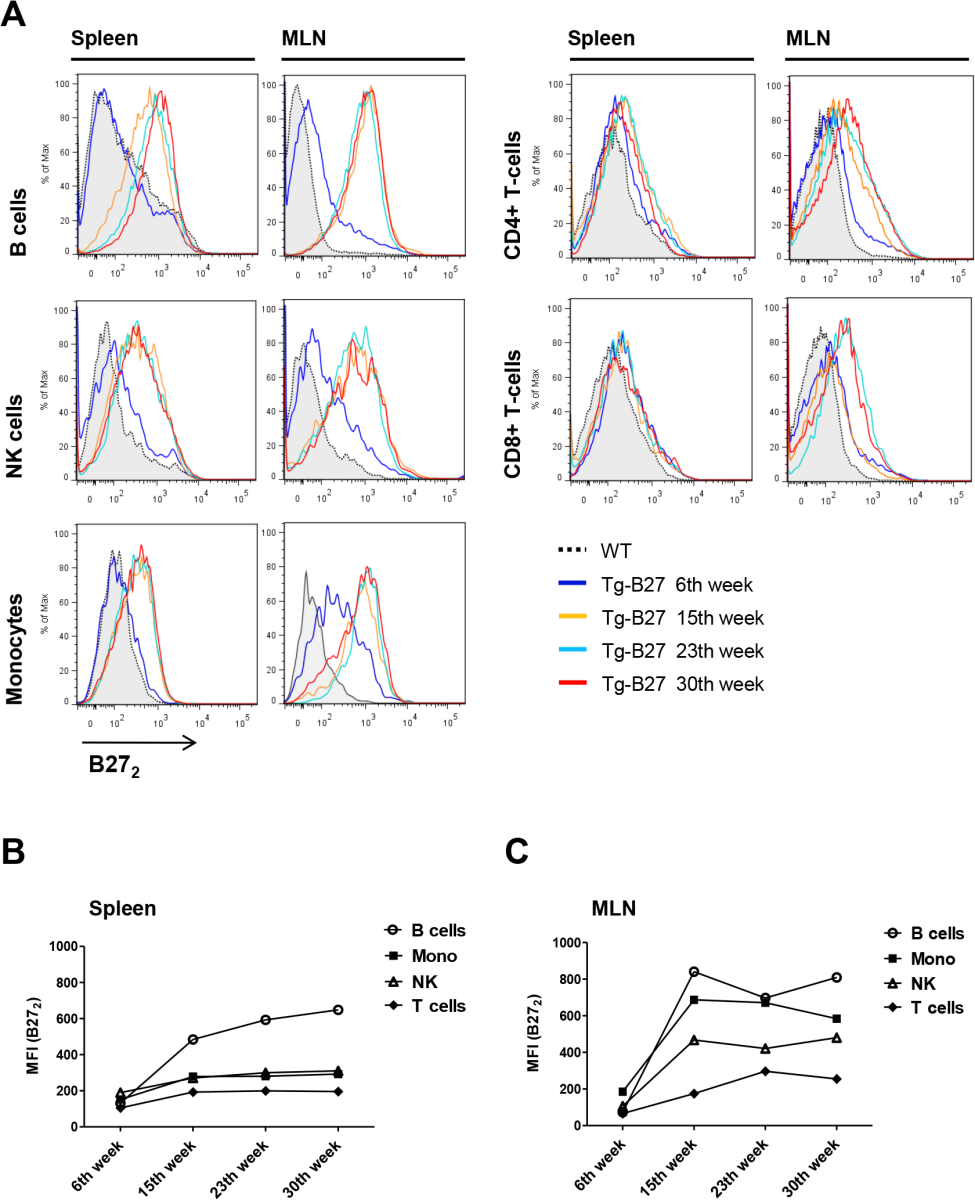
Detection of cell-surface B27_2_ in leukocyte populations of *HLA-B27* transgenic rats at different ages. **(A)** Representative flow cytometry analysis of cell-surface B27_**2**_ expression in leukocytes populations of Tg rats at different ages (6, 15, 23 and 30 weeks) from spleen and MLNs. WT leukocytes represent the control population where B27_**2**_ is absent. **(B)** MFI values plotted of positive B27_**2**_ stains from splenocytes. **(C)** MFI values plotted of positive B27_**2**_ stains from MLNs. Detection of cell-surface B27_**2**_ homodimers was performed using HD6-biotinylated and detected by streptavidin-APC. HD6 had been previously assessed as an antibody capable of recognizing cell-surface B27_**2**_ in human [12] and rat [41] leukocyte populations. Antibody panels: CD4+ T-cells (+CD3, +CD4), CD8+ T-cells (+CD3, +CD8), NK (+CD161a,—lineage), B cells (+CD45RA,-lineage) and Monocytes (+CD172a,—RP-1).

We next examined the presence of cell-surface B27_2_ in leukocyte populations in Tg rats at 6, 15, 23, and 30 weeks of age, to determine the different time points of B27_2_ expression and relation to disease progression. Results showed positive staining for B27_2_ in all leukocyte populations examined except granulocytes (Fig 4A). Cell-surface B27_2_ was almost absent in the spleen of young rats (6 weeks), but increased with age reaching a maximum between 15 and 23 weeks (Fig 4A and 4B). Lymphocytes from the draining lymph nodes of the gut (mesenchymal lymph nodes (MLN)) of Tg rats where inflammatory processes are associated to colitis displayed higher numbers of B27_2_ dimers as early as 6 weeks, and increased with age at higher rates when compared to splenocytes (Fig 4A–4C).

### Increased body weight gain in Tg-HD5 rats

**Fig 5:**
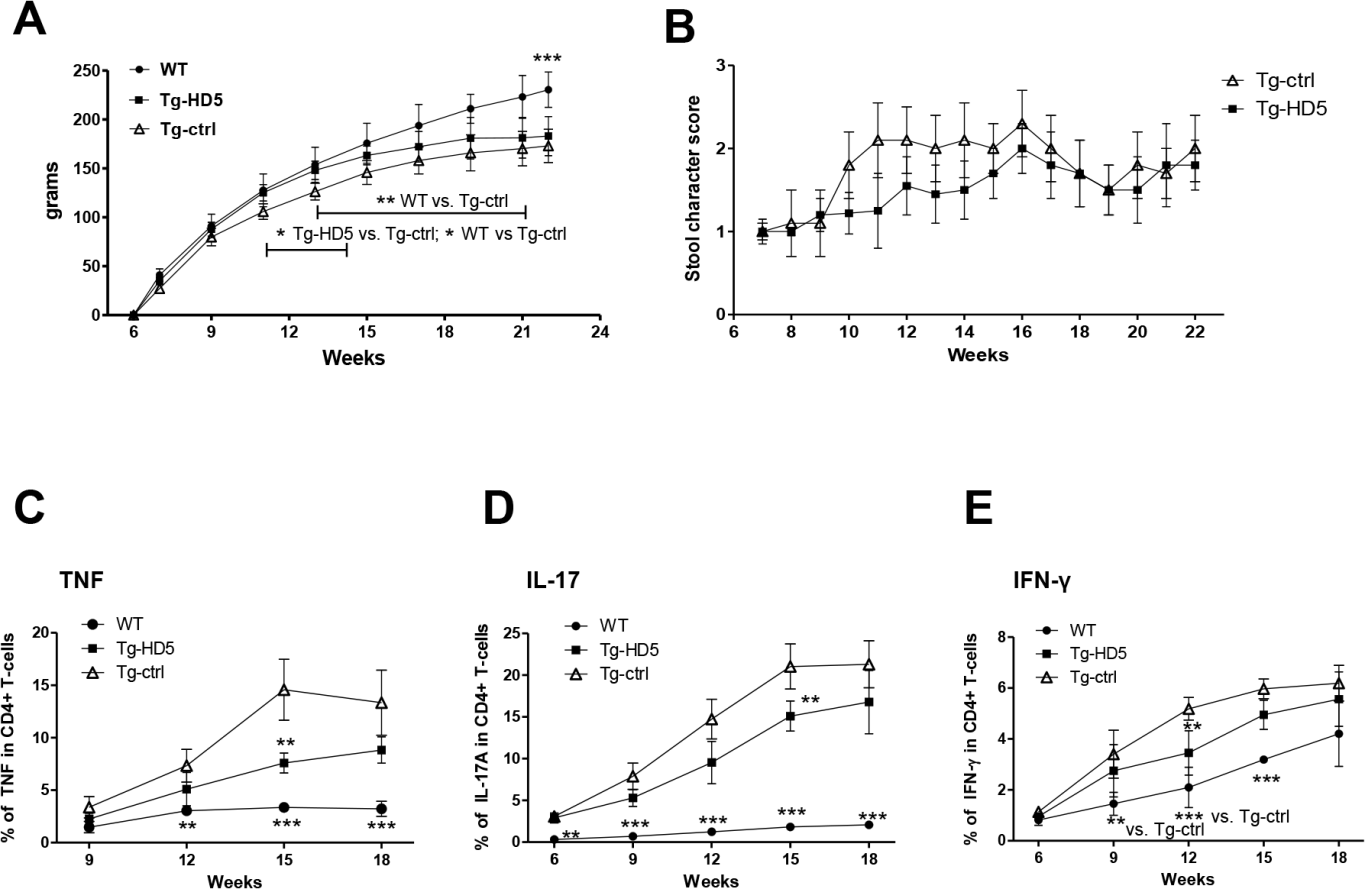
Increased body weight and decreased expansion of pro-inflammatory CD4+ T-cells from blood in Tg-HD5 rats. **(A)** Body weight gain from the 6^th^ week of age where the first antibody injections were performed. **(B)** Stool score from Tg-HD5 and Tg-ctrl groups. **(C-E)** *In vitro* stimulated cells obtained at different time points from the blood of Tg-HD5, Tg-ctrl and WT-littermates were assessed by ICS for the presence of pro-inflammatory cells expressing TNF **(C)**, IL-17 **(D)** and IFN-γ **(E)**. Results are expressed as percentages of CD4+ T gated cells. Values are expressed as mean±SEM. *p<0.05, **p<0.01, ***p<0.005 as determined by one-way ANOVA followed by Bonferroni post-hoc analysis.

Based on our data *in vitro* we hypothesized that HD5 could modulate B27_2_-dependent pro-inflammatory responses *in vivo*. Therefore, treatment with repetitive injections of HD5 in Tg rats was evaluated in a preventive setting (6 weeks of age). Body weight gain (Fig 5A) and presence of diarrhea (Fig 5B) was measured as an *in vivo* surrogate marker of B27-dependent colitis activity. *HLA-B27*/hβ2m transgenic (33–3 line) rats in our animal facility did not develop any other clinical symptoms described for this animal model [13, 16].

Onset of diarrhea in Tg-ctrl rats began between 9 to 10 weeks of age and reached a plateau at 11 weeks of age (Fig 5B), followed with significant weight loss from week 11 to 14 when compared to Tg-HD5 (p<0.05) (Fig 5A). Tg-ctrl rats were significantly different in weight from WT-littermates from week 11 till the end of the experiment (p<0.05–0.001) (Fig 5A). Onset of diarrhea in Tg-HD5 rats began between 11–14 weeks and reached a plateau between 15 to 16 weeks of age (Fig 5B). Tg-HD5 rats compared to control WT-littermates did not differ significantly in weight until week 23 (Fig 5A).

### Reduced expansion of pro-inflammatory cells in Tg-HD5 rats

During therapy, blood samples were monitored for the expansion of pro-inflammatory CD4+ T-cells expressing TNF, IL-17, and IFN-γ. Tg-HD5 rats showed a significant decrease of CD4+ T-cell expressing TNF and IL-17 at week 15 (Fig 5C and 5D). The proportion of Th1 cells producing IFN-γ remained low in Tg groups and decreased at week 12 in Tg-HD5 rats (Fig 5E).

**Fig 6:**
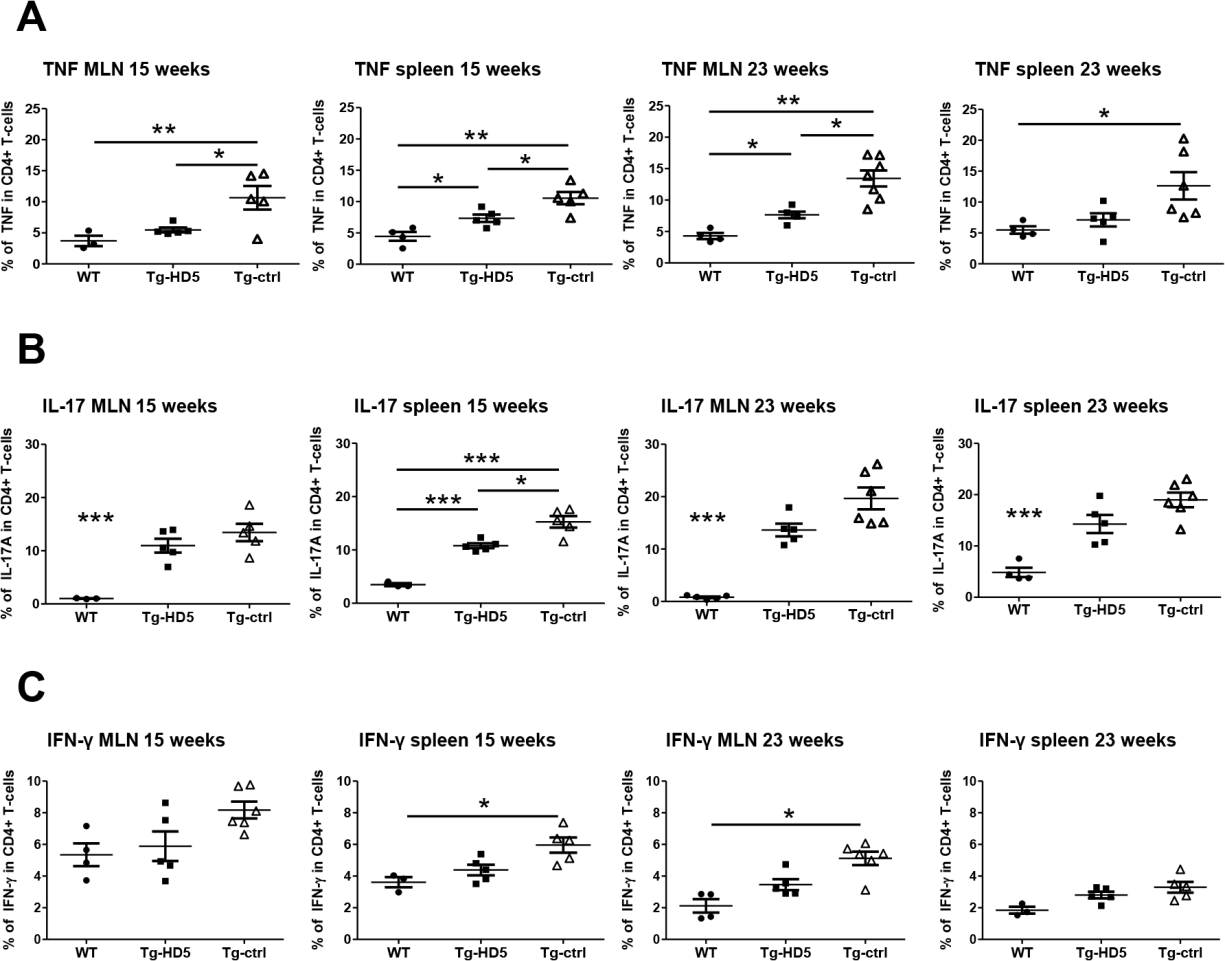
Reduced expansion of pro-inflammatory CD4+ T-cells from spleen and MLN in Tg-HD5 rats. *In vitro* stimulated cells obtained from Tg-HD5, Tg-ctrl and WT-littermates were assessed by ICS for the presence of pro-inflammatory cells expressing TNF **(A)**, IL-17 **(B)** and IFN-γ **(C)**. MLN and spleens cells were obtained at week 15 (n = 5) and at week 23 (n = 5). Results are plotted as the percentage of CD4+ T-cells gated positive for TNF, IL-17 and IFN-γ. Values are expressed as mean±SEM. *p<0.05, **p<0.01, ***p<0.005 as determined by one-way ANOVA followed by Bonferroni post-hoc analysis.

Spleen and MLNs were analyzed for the expansion of pro-inflammatory CD4+ T-cells from rats at 15 and 23 weeks of age. HD5 therapy had a significant impact on the number of CD4+ T-cells producing TNF from splenocytes at 15 weeks, and MLNs at 15 and 23 weeks (Fig 6A), respectively, and strongly correlated with the numbers of TNF+ CD4+ T-cells circulating in the blood (Fig 5C). The number of Th17 cells was reduced in the spleen of Tg- HD5 rats at week 15, but not at week 23 (Fig 6B). The number of Th1 cells under HD5 mAb therapy was not significantly different between Tg groups (Fig 6C), however, Th1 cells from Tg-ctrl were significantly elevated compared to WT rats at week 15 and week 23.

### Immunohistochemistry

Histopathological scoring analysis evidenced that colonic mucosa presented infiltration of inflammatory cells in Tg rats at 15 and 23 weeks (S5 Fig). No significance between groups was observed between groups at week 15 Tg-HD5 (1.7 ± 0.54) and Tg-ctrl (2.35 ± 0.65) p<0.079, and week 23 Tg-HD5 (2.54 ± 0.43) and Tg-ctrl (2.46 ± 0.62). The histological score for WT-littermates (0.25 ± 0.18) was significantly decreased compared to *HLA-B27* rats upon treatment with both antibodies (p<0.01). No differences were found between WT and Tg groups from histological samples of ileum, duodenum and jejunum (S6–S8 Figs).

### Reduction of soluble TNF in Tg-HD5 rats

**Fig 7:**
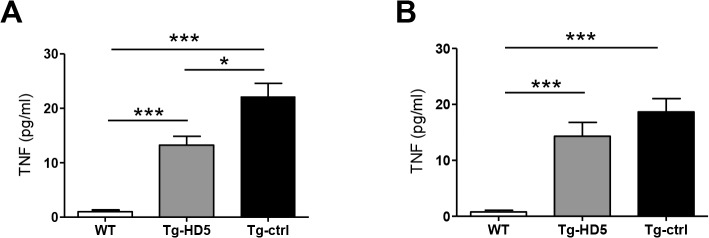
Reduced amount of soluble TNF in Tg-HD5 rats. Serum obtained from the blood after euthanasia was assessed for the presence of soluble TNF. **(A-B)** TNF serum ELISA from WT, Tg-HD5 and Tg-ctrl (n = 5 per group) at 15 weeks **(A)** and at 23 weeks **(B)**.

Serum samples were analyzed for the presence of soluble TNF. TNF levels were significantly reduced in Tg-HD5 rats at early treatment stages when compared to Tg-ctrl (13.2 pg/mL vs. 22.13 pg/mL, respectively, Fig 7A), but not at later time points (14.3 pg/mL vs. 18.6 pg/mL, respectively, Fig 7B). Serum IL-17 levels measured by ELISA were below detection limit and, therefore, not determined.

### Cell-surface B27_2_ is reduced in Tg-HD5 rats

**Fig 8:**
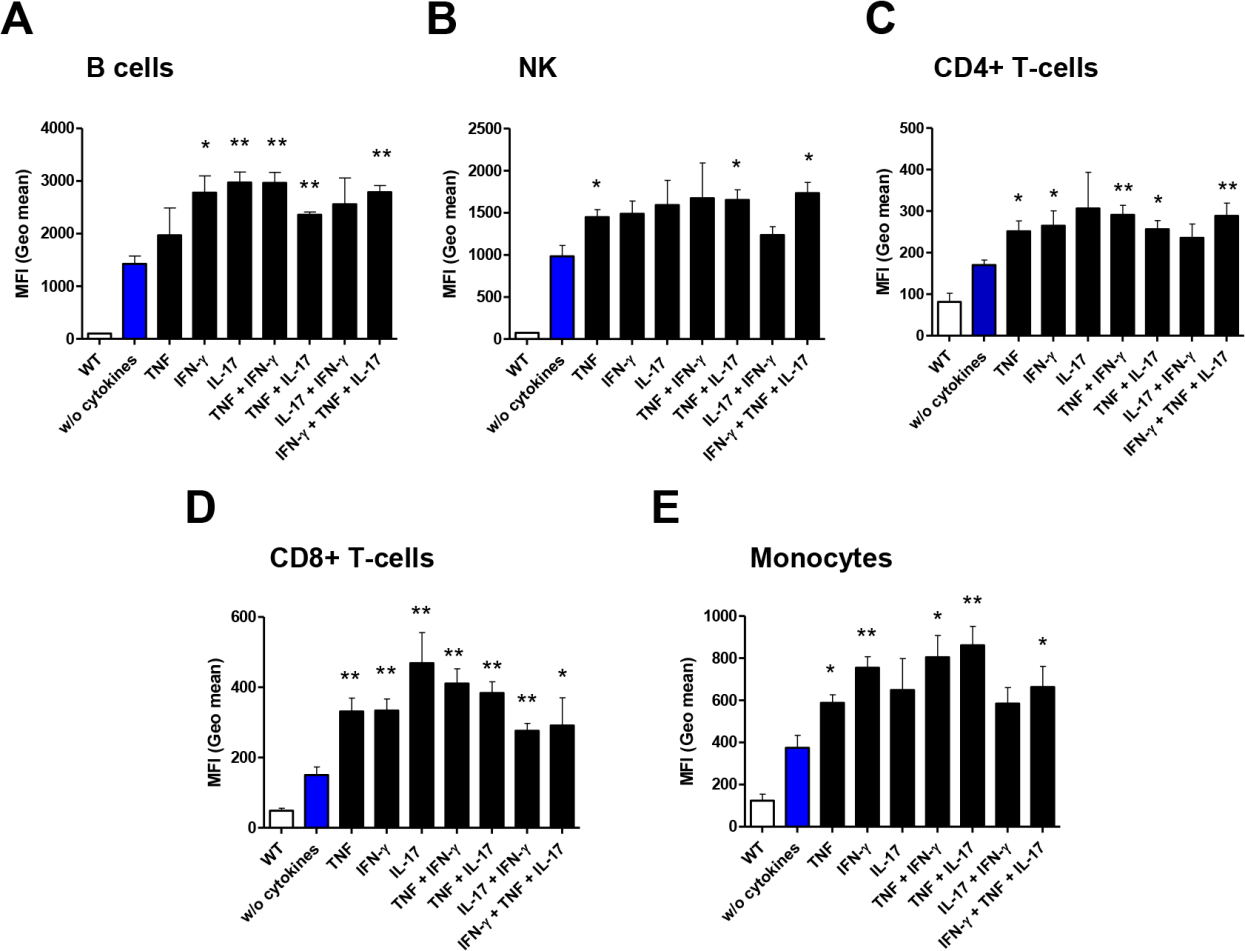
Pro-inflammatory cytokines increase cell-surface B27_2_ homodimers. **(A-E)** Cell cultures of leukocyte populations were analyzed by flow cytometry for presence of cell-surface B27_**2**_ homodimers after stimulation with cytokines. HD6-biotinylated and streptavidin APC were used for detection. Mean fluorescence intensity (MFI) values were plotted from data analysis. Statistical analysis was plotted against “without cytokines” data values. Values are expressed as mean±SEM. *p<0.05, **p<0.01, ***p<0.005 as determined by one-way ANOVA followed by Bonferroni post-hoc analysis.

We then sought to better understand the role of pro-inflammatory cytokines in the induction of cell-surface B27_2_. TNF and IFN-γ have been previously shown to induce the gene synthesis of B27 [38], and to increase the intracellular accumulation of B27_2_ homodimers in leukocytes and transfected cells [39]. Soluble cytokines alone or in combinations (TNF, IFN-γ, and IL-17) significantly induced the accumulation of cell-surface B27_2_ molecules on rat leukocytes after *in vitro* exposure (Fig 8A–8E), suggesting that a pro-inflammatory milieu could influence the amount of B27_2_ molecules *in vivo*.

### B27_2_ cell-surface homodimers are reduced in leukocytes of HD5-treated rats

**Fig 9:**
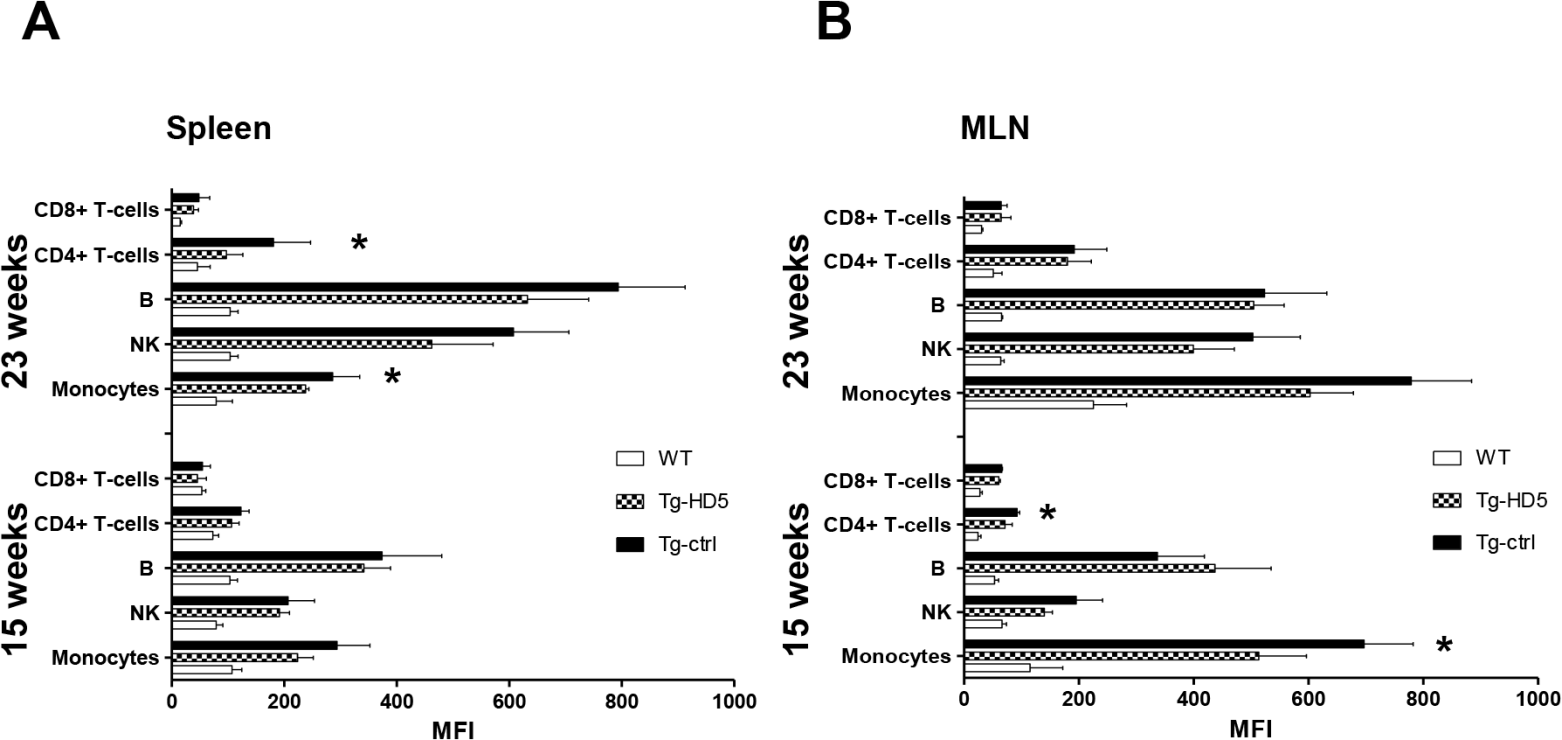
Reduced accumulation of cell-surface B27_2_ molecules in Tg-HD5 rats. Analysis of cell-surface B27_**2**_ from spleen and MLN was performed using HD6-biotinylated and detected by streptavidin-APC in WT-littermates and *HLA-B27* rat groups at 15 and 23 weeks**. (A-B)** MFI values plotted of positive B27_**2**_ stains from flow cytometry analyzed splenocytes (n = 5) **(A)** and MLN (n = 5) **(B)** subpopulations. Values are expressed as mean±SEM. *p<0.05, ***p<0.005 as determined by one-way ANOVA followed by Bonferroni post-hoc analysis.

To assess if the number of B27_2_ molecules changed during therapy in Tg-HD5 rats, we determine the amount of cell-surface B27_2_ using MFI values. We used the HD6 antibody for staining of leukocytes to avoid any potential ex vivo staining overlap with Tg-HD5 treated rats. HD5 and HD6 do not compete for the same epitope of B27_2_ (S1 Fig). Results showed that HD5 treated rats have reduced amounts of cell-surface B27_2_ and that the most influenced type of cells were monocyte populations (Fig 9A and 9B). Other groups of leukocytes such as CD4+ T-cells seem to be also influenced by the HD5 mAb treatment, suggesting that the amount of cell-surface B27_2_ can be controlled by processes triggered from extracellular pro-inflammatory signals.

## Discussion

The presence of cell-surface B27_2_ in leukocytes from patients and animal models of SpA led to the hypothesis that active disease is triggered by the immune recognition of B27_2_ molecules to lymphoid and/or myeloid immune cells, resulting in inflammatory responses [37]. B27_2_ has been previously reported to interact specifically to human KIR3DL2, and to human LILRB2 and mouse Pirb in a manner different from correctly folded HLA-B27 complexes [11, 37]. Therefore, we postulated that antibodies blocking the interaction of B27_2_ dimers to corresponding immune-receptors could potentially modify disease activity and inflammatory responses in *HLA-B27* transgenic rats.

The first reagent tested against cell-surface B27_2_ in a murine model of SpA was the HC10 antibody. Results demonstrated that a therapeutic approach using HC10 ameliorated disease progression in this mouse model [40]. However, disadvantages of using the HC10 antibody as a therapeutic agent relates to its unspecificity, as it interacts with all other classes of β2m-free heavy chains of HLA-B and-C alleles, including both B27_2_ and B27-free heavy chain forms.

Here, we describe a novel antibody (HD5) against B27_2_ derived from a phage display library. We showed that the HD5 antibody displays greater specificity towards B27_2_ when compared to our previously described HD6 [12], and HC10 antibodies. Furthermore, HD5 was able to block the interactions of B27_2_ to its known leukocyte receptors (KIR3DL2, LILRB2 and mouse Pirb) as shown by SPR. Additionally, *In vitro* assays demonstrated that B27_2_ interaction with CD4+ T-cells induced pro-inflammatory responses (secretion of TNF) from both Tg and WT cells, and that blocking these interactions by pre-incubating HD5 with B27_2_ resulted in decreased production of TNF.

In HLA-B27 rats we first studied the cell-surface expression of B27_2_ on lymphocyte populations at different time points in spleen and MLN to determine the feasibility of targeting B27_2_ with HD5. B272 staining of was consistent with data of our previous publication [41] and allowed for a more accurate definition of various time points. We showed that a dramatic increase of B27_2_ expression occurred on B cells, NK cells and monocytes between 6 and 15 weeks of age. Interestingly, elevated levels of cell-surface B27_2_ reached a threshold between 15 to 23 weeks and correlated with the stabilization of pro-inflammatory CD4+ T-cells expansion and with colitis severity. Additionally, B27_2_ was shown to be more abundant at the draining lymph nodes of the gut where inflammatory processes are strongly associated with expansion of Th17, Th1 and CD4+ TNF+ T-cells. Only MLNs but not splenocytes from 6 weeks old rats displayed cell-surface B27_2_ in monocytes together with increased Th17 expansion, indicating that inflammatory processes began earlier in life before any clinical symptom appeared. Taurog *et al* previously demonstrated that disease susceptibility in HLA-B27 rats correlated with the B27 transgene copy number, and that surface expression of HLA-B27 heterotrimers increased with age and disease onset [42]. These results point out to a model were increased expression of HLA-B27 correlates with a higher number of cell-surface B27_2_ molecules and with disease onset and/or progression. Also these data suggest that a progressive increase of B27_2_ is consistently related to inflammatory responses, at least in *HLA-B27* rats. In PBMCs of *HLA-B27*+ AS patients we have previously demonstrated the presence of cell-surface B27_2_ in monocytes [12], however, its involvement in disease has not yet been clarified.

HLA-B27 associated disease progression in rats was accompanied by the expansion of pro-inflammatory CD4+ T-cells expressing IL-17, TNF and IFN-γ [15] as early as 6 weeks of age, and clinical symptoms (colitis) appeared at approximate 10 weeks of age. Disease establishment took place at ~15 weeks old animals when the expansion of CD4+ T-cells expressing IL-17, TNF and IFN-γ reached a threshold together with stabilization of colitis severity and accumulation of B27_2_ molecules. We did not observe any other clinical symptoms described for this animal model such as arthritis, psoriasis or nail dystrophy. Environmental factors are known to be key determinants to the onset of clinical pathology of HLA-B27 rats [16]. Experiments comparing germ free transgenic rats, transgenic rats under non-SPF conditions and transgenic rats colonized with defined bacterial cocktails have demonstrated that the gut flora is key to the onset and progression of clinical symptoms [43, 44]. Therefore, variations in disease progression can be observed by the hygiene status of the animal facility.

Antibody therapy with HD5 at early stages of the treatment (15 weeks) was accompanied with reduced soluble total TNF, decreased CD4+ T-cell numbers expressing TNF from blood, spleen and MLNs. These data were in accordance to our *in vitro* observations, where addition of HD5 to either recombinant B27_2,_ or cell-surface B27_2_ presented by .220 B27 cells blocked pro-inflammatory TNF responses in CD4+ T-cells. *In vivo*, although we observed that TNF production was modulated by HD5 we could only determine trends by histological scoring of the colon. Previous studies from Milia *et al* have shown that anti-TNF therapy using monoclonal antibodies does not modify histological disease progression of the colon in this *HLA-B27* rat model where pathology has been established at >18 weeks, and exhibit only benefit for the onset of disease [34, 45]. In contrast, TNF blockage in AS patients is an effective therapeutic approach able to show improvement of disease activity in 60% of patients with active spondylitis. We could also determine that B27_2_ did not induce IL-17 production in naïve CD4+ T-cells *in vitro*. However, induction of IL-17 in +KIR3DL2 CD4+ T-cells by B27_2_ has been demonstrated in AS patients [7]. It is plausible that interactions of B27_2_ with subpopulation of CD4+ T-cells in rats may enhance the production of IL-17. Unfortunately, an anti-KIR3DL2 homologue reagent for rat has yet not been described and experiments of this type were not conducted. HD5 antibody therapy partially modulated the cell expansion of Th17, however, we observed that expansion of these cells was significantly elevated throughout the experiment when compared to WT. We assume that a combination of factors may have a direct effect on the expansion of Th17 cells. For example, gut inflammation in this rat model has been linked to UPR responses and to production of IL-23 in rats [46, 47]. IL-23, apart from inducing the expression of IL-17, can also exacerbate spondyloarthirits type symptoms in entheseal lymphocytes as demonstrated in a SpA mouse model [48]. Other factors such as over-expression of *HLA-B27* (55 gene copies per cell) could lead to intracellular accumulation of B27_2_ and, therefore, to unfolded protein responses (UPR) in different cell types. Therefore, therapeutic strategies targeting cell-surface B27_2_ homodimers using antibodies may be hampered as a therapeutic read out. The use of an alternative animal model with a broad spectrum of SpA clinical symptoms [49] needs to be further explored.

Other factors that may diminish the effect of HD5 antibody therapy may be related to the development of neutralizing antibodies against HD5. This effect is well known and described in several models [50]. An alternative explanation for reduced therapeutic effect could be the limited penetration of HD5 to the mucosa of the gut especially during inflammation, this problem is well described for anti-cancer antibodies [51].

Throughout therapy, HD5 was assessed as a potential depleting antibody. Flow cytometry analysis showed that leukocytes numbers between Tg-HD5 vs Tg-ctrl were not modified at any time point of the study (data not shown), and therefore could not influence the amount of cells presenting B27_2_

Mechanisms influencing B27_2_ expression can be related to pro-inflammatory cytokines. TNF and IFN-γ have been shown to induce the gene synthesis of *HLA-B27* [38], and to increase the intracellular protein accumulation of B27_2_ in leukocytes [39, 52]. Here we have shown that IL-17, TNF and IFN-γ independently or in combination regulate B27_2_ formation in leukocytes. We propose that exposure to an inflammatory cytokine milieu regulates B27_2_ formation, leading to a positive feedback loop that exacerbates and perpetuates the presence of B27_2_ and, therefore, favors pro-inflammatory responses. Consistent with this observation, modulation of TNF by HD5 in Tg rats resulted in reduced amounts of cell-surface B27_2_. Hence we hypothesize that soluble cytokines may influence and control the accumulation of B27_2_ in Tg animals. Analysis of cell-surface B27_2_ expression in patients with cytokine (e.g. anti-TNF,-IL-17) blockade therapy may help to clarify this particular issue and are currently under way.

Our findings indicate for the first time that targeted therapies to cell-surface B27_2_ using HD5 could delay pro-inflammatory responses *in vivo* by modulating the expansion of TNF CD4+ T-cells. The HD5 antibody described here has distinct sequence and unique specificity for B27_2_ when compared to our previously described HD6 antibody [12, 41]. Targeting B27_2_ is proposed as a potential therapeutic approach for modulating pro-inflammatory responses and together with combinatorial therapies (e.g. anti-IL-17/23 or TNF blockade) could emerge as an alternative approach for the treatment of *HLA-B27* related disorders.

### Ethics approval

Institutional Cantonal Animal Care and Use Committees (184/2011).

## Supporting Information

S1 Fig HD5, HD6 and HC10 compete for a different epitope in B27_2._
**(A)** Epitope competition experiments in SPR were performed by immobilizing HD5 to CM5 chips and binding events recorded. Next, recombinant B27_2_ was injected into the system, followed by injection of HD5 to show there was no further interaction with B27_2_. Then, injection of a second antibody (HD6), proceeded by the injection of a third antibody (HC10) show binding of both antibodies to B27_2_-HD5 complexes. **(B)** Schematic representation of antibody interaction in the epitope competition experiment. RU = responsive units.
(TIF)

S2 Fig In solution equilibrium “true affinity” of HD5. **(A)** Calibration of binding stability of B27_2_ to HD5, by employing concentration series of B27_2_ ranging from 0.01 to 1 nM in triplicates. **(B)** Fixed concentrations of 1 nM B27_2_ were incubated with varying concentrations of HD5 0.01 to 128 nM for 2h at room temperature in triplicates. In solution equilibrium reaction mixtures were analyzed for the concentration of free B27_2_ binding to the chip. RU = responsive units.
(TIF)

S3 Fig B27_2_ (1x) tetramers do not induce the production of IL-17 or IFN-γ in rat CD4+ T-cells. **(A)** Tg and WT CD4+ T-cells do not produce IL-17 after incubation with B27_2_ (1x)-tetramers. **(B)** Tg and WT CD4+ T-cells do not produce IFN- after incubation with B27_2_ (1x)-tetramers. Tests were performed in triplicates. Tet = tetramer. Values are expressed as mean±SEM. Statistical analysis was determined by one-way ANOVA followed by Bonferroni post-hoc analysis.
(TIF)

S4 Fig η cells do not induce the expression of IL-17 or IFN-γ. **(A)** .220 B27 cells do not induce the production of IL-17 in rat CD4+ T-cells. **(B)** .220 B27 cells do not induce the production of IFN-γ in rat CD4+ T-cells. Tests were performed in triplicates. Tet = tetramer. Values are expressed as mean±SEM. Statistical analysis was determined by one-way ANOVA followed by Bonferroni post-hoc analysis.
(TIF)

S5 Fig Histological scoring of H&E staining of colon. **(A)** Histological score of colon. Representative images of WT-littermates 15 weeks **(B)**, Tg-ctrl 15 weeks **(C,E),** Tg-HD5 15 weeks **(D,F),** Tg-ctrl 23 weeks **(G)** and Tg-HD5 23 weeks **(H)**. **(A-B)** WT-littermate rats showed no signs of inflammation and an intact epithelial barrier compared to a thickened mucosa and lymphocyte influx in *HLA-B27* rats. *HLA-B27* rats showed intact crypts without damage to intestinal epithelial cells **(C-H)**. Tg rats showed thickening of the mucosa in large areas. Goblet cells were present in the expected number **(E-F).** Images are representative for 5 rats each. White arrows indicate areas of lymphocyte influx. Orange arrows indicate presence of goblet cells. # indicates the lamina muscularis mucosae. Original magnification **(B-D,G and H)** 5-fold, **(E-F)** 20-fold. Values are expressed as mean±SEM. ***p<0.005 as determined by one-way ANOVA followed by Bonferroni post-hoc analysis.
(TIF)

S6 Fig Histological scoring and H&E staining of jejunum. **(A)** Histological score of jejunum. Representative images of WT-littermates 23 weeks **(B)**, Tg-ctrl 15 weeks **(C),** Tg-HD5 15 weeks **(D),** Tg-ctrl, 23 weeks **(E)** and Tg-HD5 23 weeks **(F)**. **(A-F)** No differences were observed between animal groups. Images representative for 5 rats each. Arrows indicate area with an increased number of lymphocytes. Original magnification 5-fold. Values are expressed as mean±SEM.
(TIF)

S7 Fig Histological scoring and H&E staining of ileum. **(A)** Histological score of ileum. Representative images of WT-littermates 23 weeks **(B)**, Tg-ctrl 15 weeks **(C),** Tg-HD5 15 weeks **(D),** Tg-ctrl, 23 weeks **(E)** and Tg-HD5 23 weeks **(F)**. **(A-F)** No differences were observed between animal groups. Images representative for 5 rats each. White arrows indicate areas of a Peyer’s patch and lymphocyte influx. Original magnification 5-fold. Values are expressed as mean±SEM.
(TIF)

S8 Fig Histological scoring and H&E staining of duodenum. **(A)** Histological score of duodenum. Representative images of WT-littermates 23 weeks **(B)**, Tg-ctrl 15 weeks **(C),** Tg-HD5 15 weeks **(D),** Tg-ctrl, 23 weeks **(E)** and Tg-HD5 23 weeks **(F)**. **(A-F)** No differences were observed between animal groups. Images representative for 5 rats each. White arrows indicate areas of lymphocyte influx. Original magnification 5-fold. Values are expressed as mean±SEM.
(TIF)
