# Supplementary figures and images for: HLA-B27-Homodimer-Specific Antibody Modulates the Expansion of Pro-Inflammatory T-Cells in HLA-B27 Transgenic Rats
Source: PLoS One. 2015 Jun 30;10(6):e0130811. doi: 10.1371/journal.pone.0130811 (PMC4488392; doi:10.1371/journal.pone.0130811)

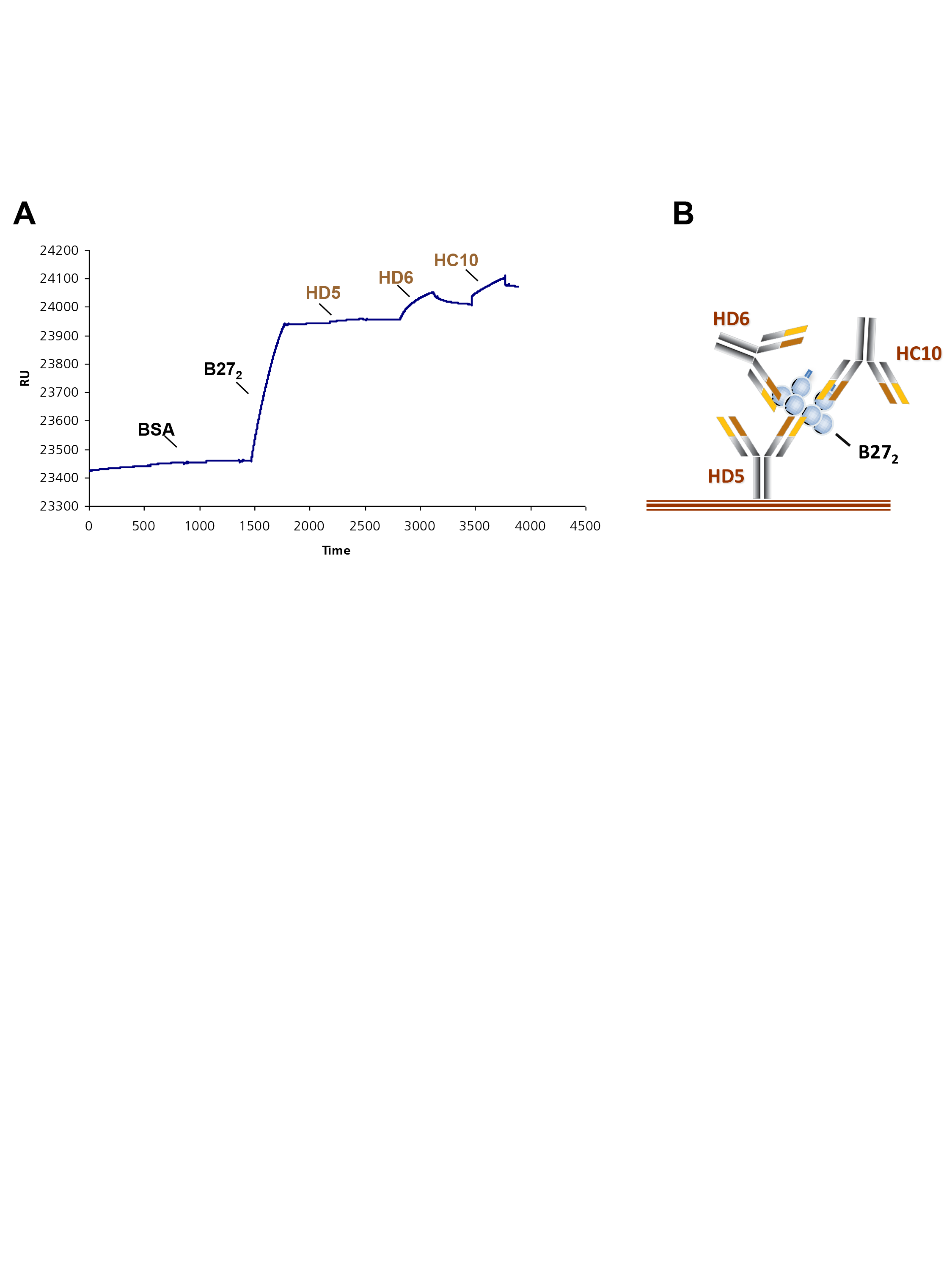

Supplement: S1 Fig — (A) Epitope competition experiments in SPR were performed by immobilizing HD5 to CM5 chips and binding events recorded. Next, recombinant B272 was injected into the system, followed by injection of HD5 to show there was no further interaction with B272. Then, injection of a second antibody (HD6), proceeded by the injection of a third antibody (HC10) show binding of both antibodies to B272-HD5 complexes. (B) Schematic representation of antibody interaction in the epitope competition experiment. RU = responsive units. (TIF) [file pone.0130811.s001.tif]

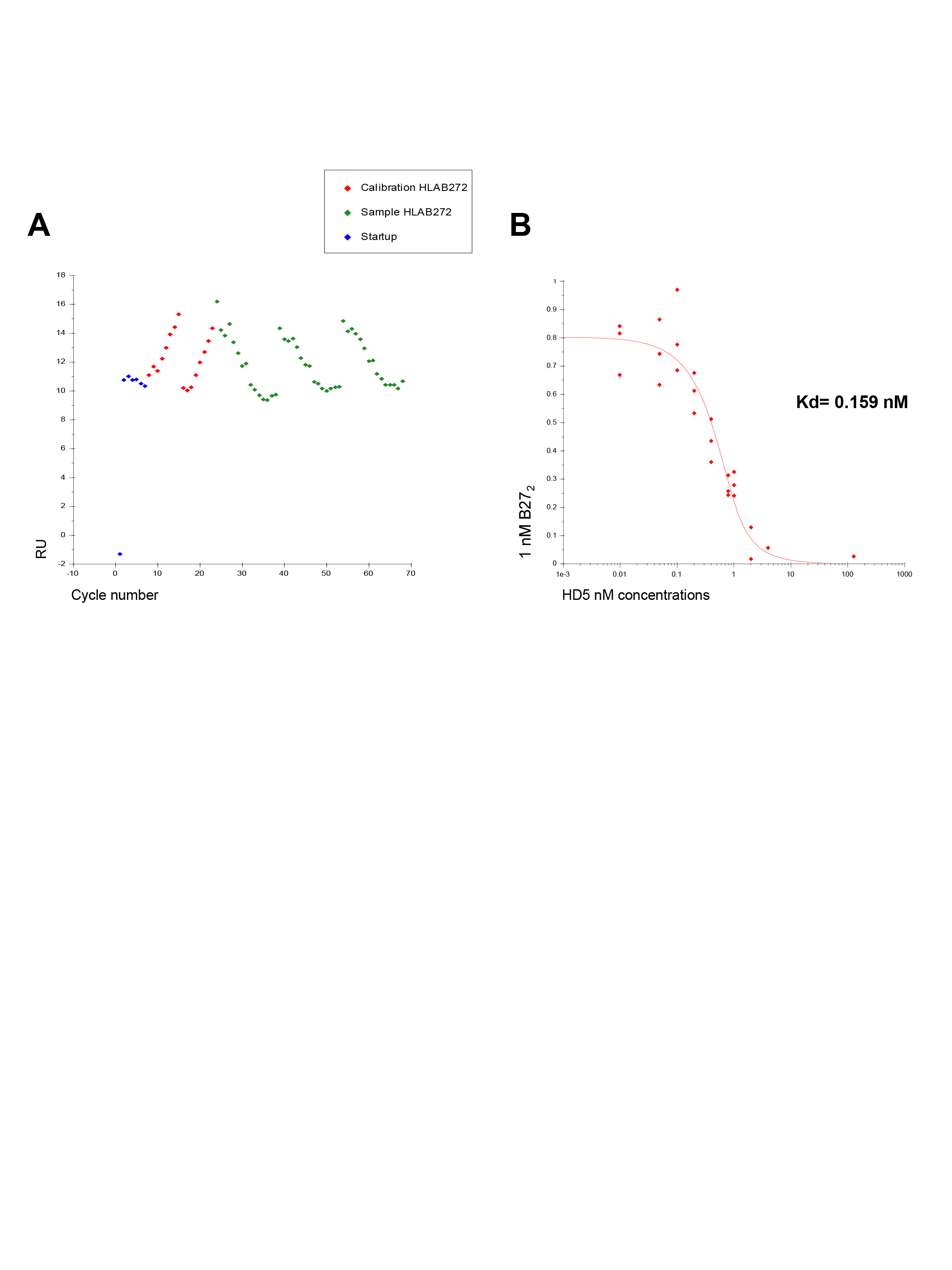

Supplement: S2 Fig — (A) Calibration of binding stability of B272 to HD5, by employing concentration series of B272 ranging from 0.01 to 1 nM in triplicates. (B) Fixed concentrations of 1 nM B272 were incubated with varying concentrations of HD5 0.01 to 128 nM for 2h at room temperature in triplicates. In solution equilibrium reaction mixtures were analyzed for the concentration of free B272 binding to the chip. RU = responsive units. (TIF) [file pone.0130811.s002.tif]

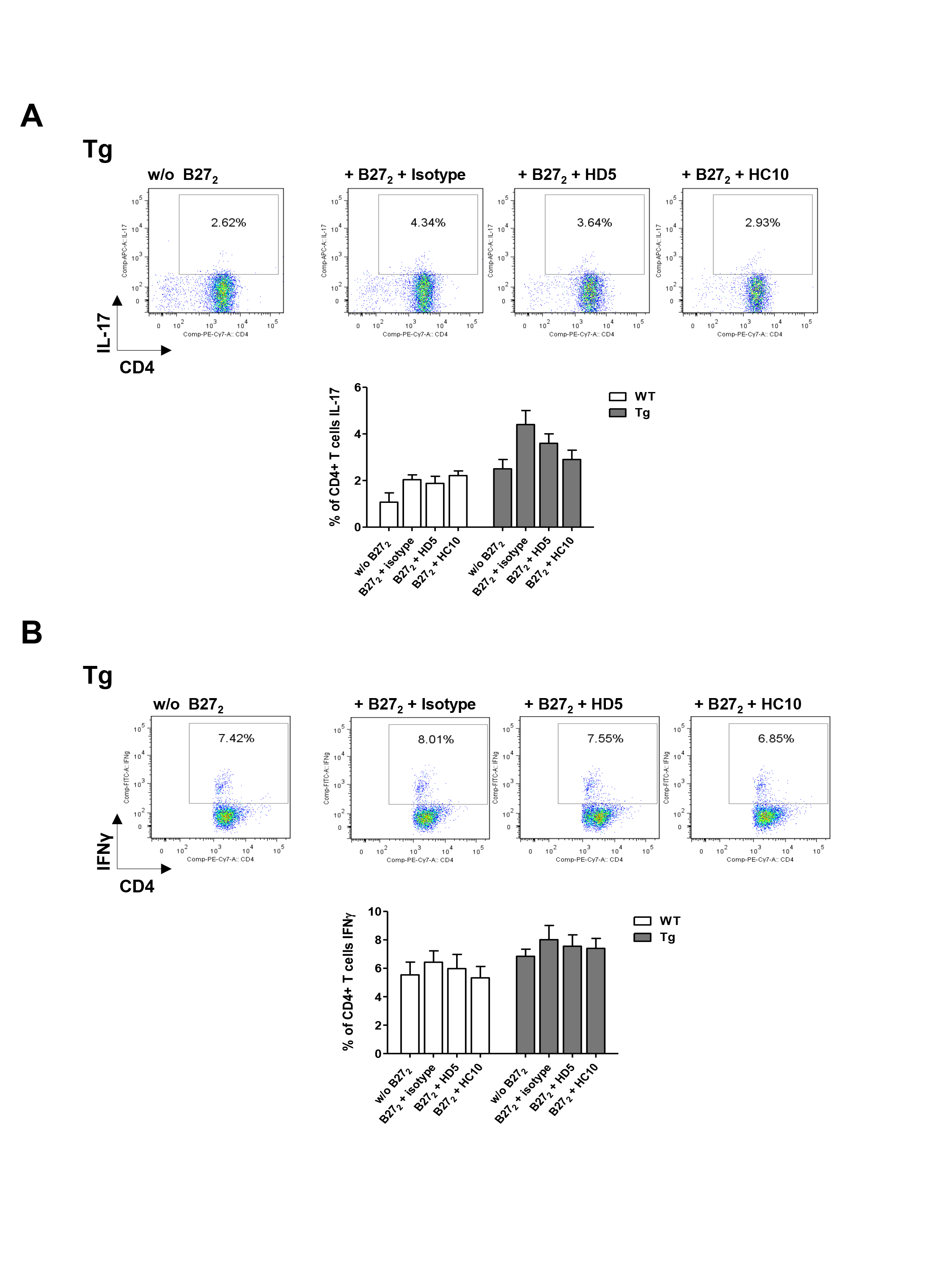

Supplement: S3 Fig — (A) Tg and WT CD4+ T-cells do not produce IL-17 after incubation with B272 (1x)-tetramers. (B) Tg and WT CD4+ T-cells do not produce IFN- after incubation with B272 (1x)-tetramers. Tests were performed in triplicates. Tet = tetramer. Values are expressed as mean±SEM. Statistical analysis was determined by one-way ANOVA followed by Bonferroni post-hoc analysis. (TIF) [file pone.0130811.s003.tif]

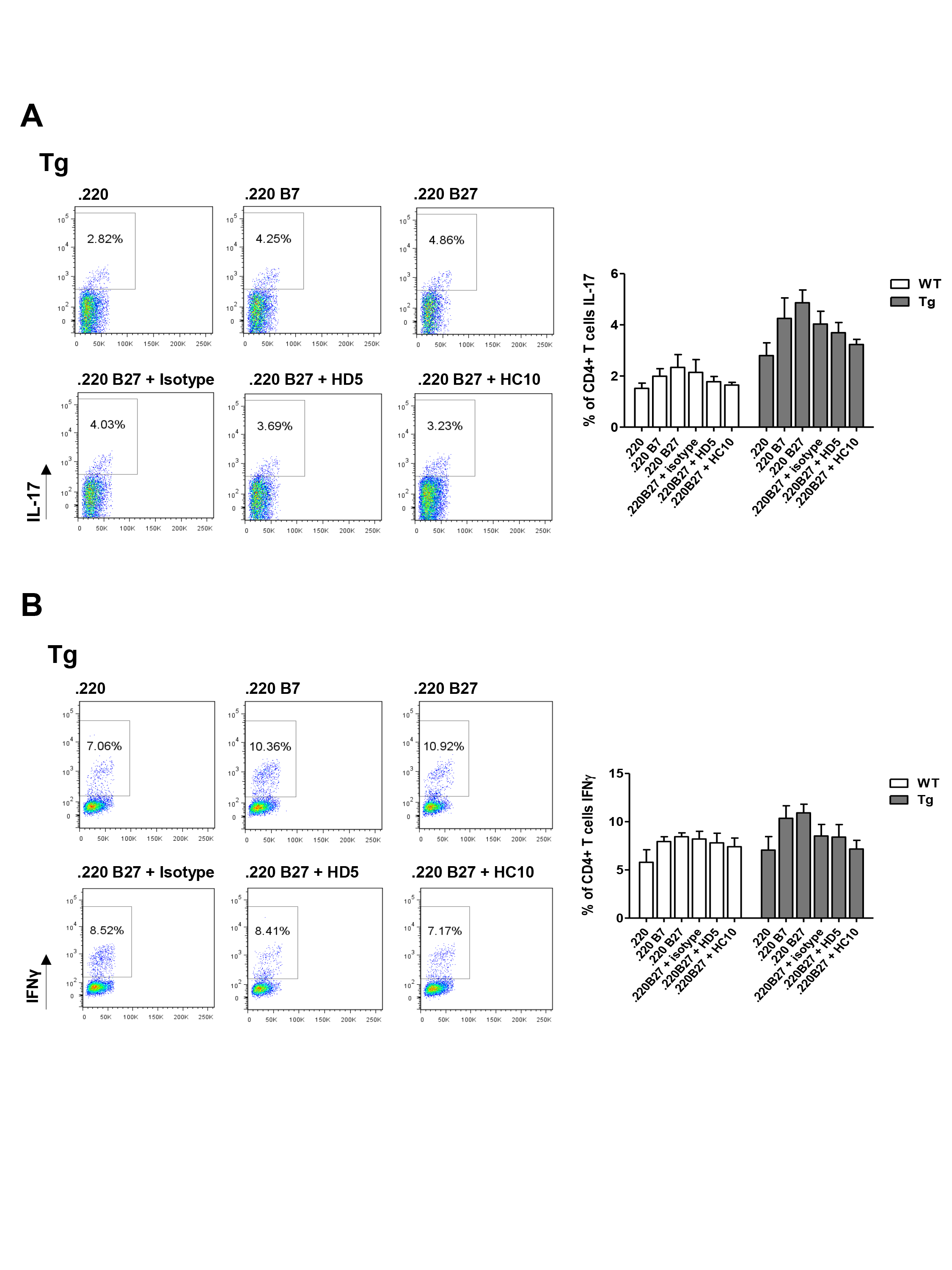

Supplement: S4 Fig — (A) .220 B27 cells do not induce the production of IL-17 in rat CD4+ T-cells. (B) .220 B27 cells do not induce the production of IFN-γ in rat CD4+ T-cells. Tests were performed in triplicates. Tet = tetramer. Values are expressed as mean±SEM. Statistical analysis was determined by one-way ANOVA followed by Bonferroni post-hoc analysis. (TIF) [file pone.0130811.s004.tif]

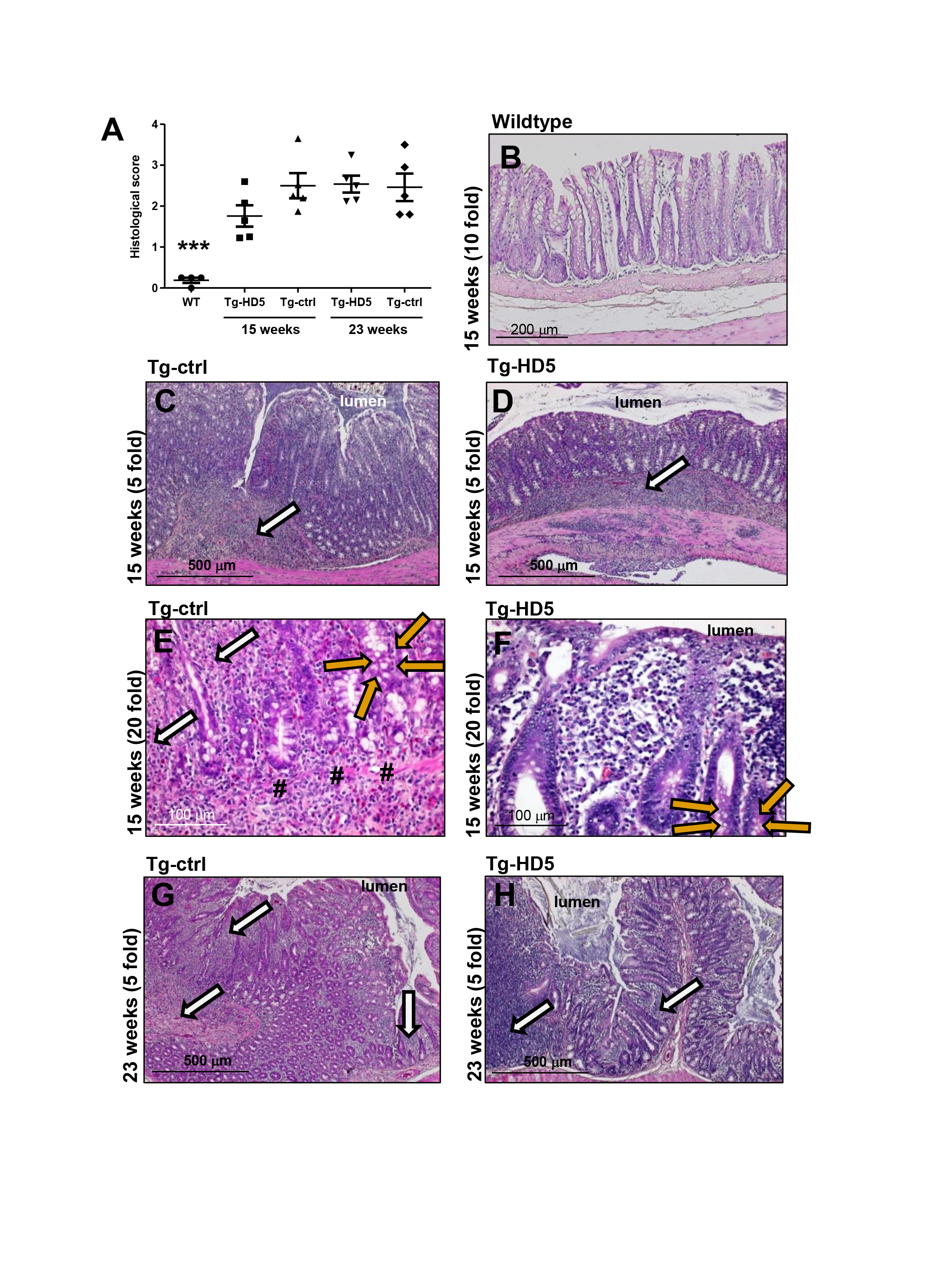

Supplement: S5 Fig — (A) Histological score of colon. Representative images of WT-littermates 15 weeks (B), Tg-ctrl 15 weeks (C,E), Tg-HD5 15 weeks (D,F), Tg-ctrl 23 weeks (G) and Tg-HD5 23 weeks (H). (A-B) WT-littermate rats showed no signs of inflammation and an intact epithelial barrier compared to a thickened mucosa and lymphocyte influx in HLA-B27 rats. HLA-B27 rats showed intact crypts without damage to intestinal epithelial cells (C-H). Tg rats showed thickening of the mucosa in large areas. Goblet cells were present in the expected number (E-F). Images are representative for 5 rats each. White arrows indicate areas of lymphocyte influx. Orange arrows indicate presence of goblet cells. # indicates the lamina muscularis mucosae. Original magnification (B-D,G and H) 5-fold, (E-F) 20-fold. Values are expressed as mean±SEM. ***p<0.005 as determined by one-way ANOVA followed by Bonferroni post-hoc analysis. (TIF) [file pone.0130811.s005.tif]

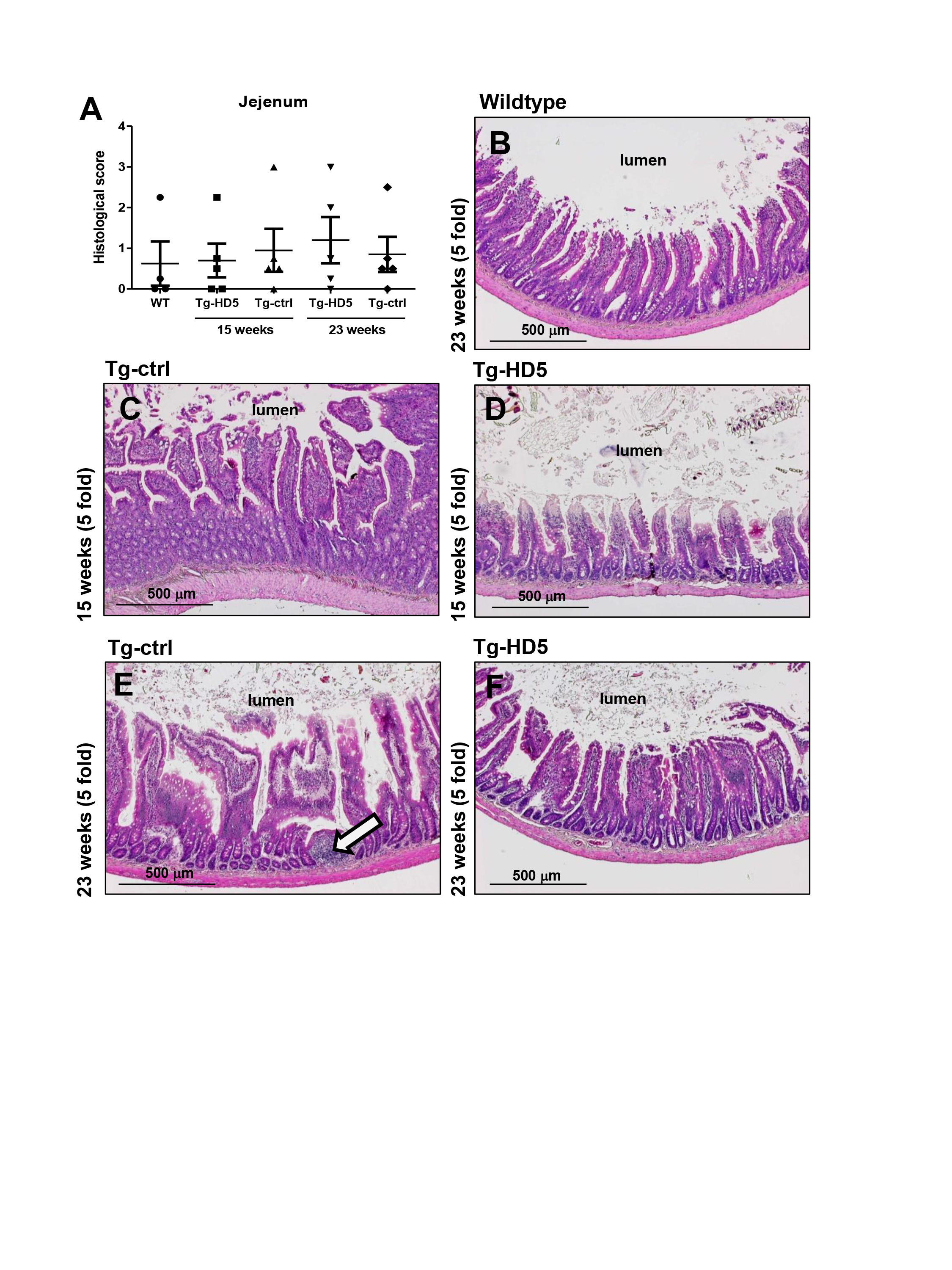

Supplement: S6 Fig — (A) Histological score of jejunum. Representative images of WT-littermates 23 weeks (B), Tg-ctrl 15 weeks (C), Tg-HD5 15 weeks (D), Tg-ctrl, 23 weeks (E) and Tg-HD5 23 weeks (F). (A-F) No differences were observed between animal groups. Images representative for 5 rats each. Arrows indicate area with an increased number of lymphocytes. Original magnification 5-fold. Values are expressed as mean±SEM. (TIF) [file pone.0130811.s006.tif]

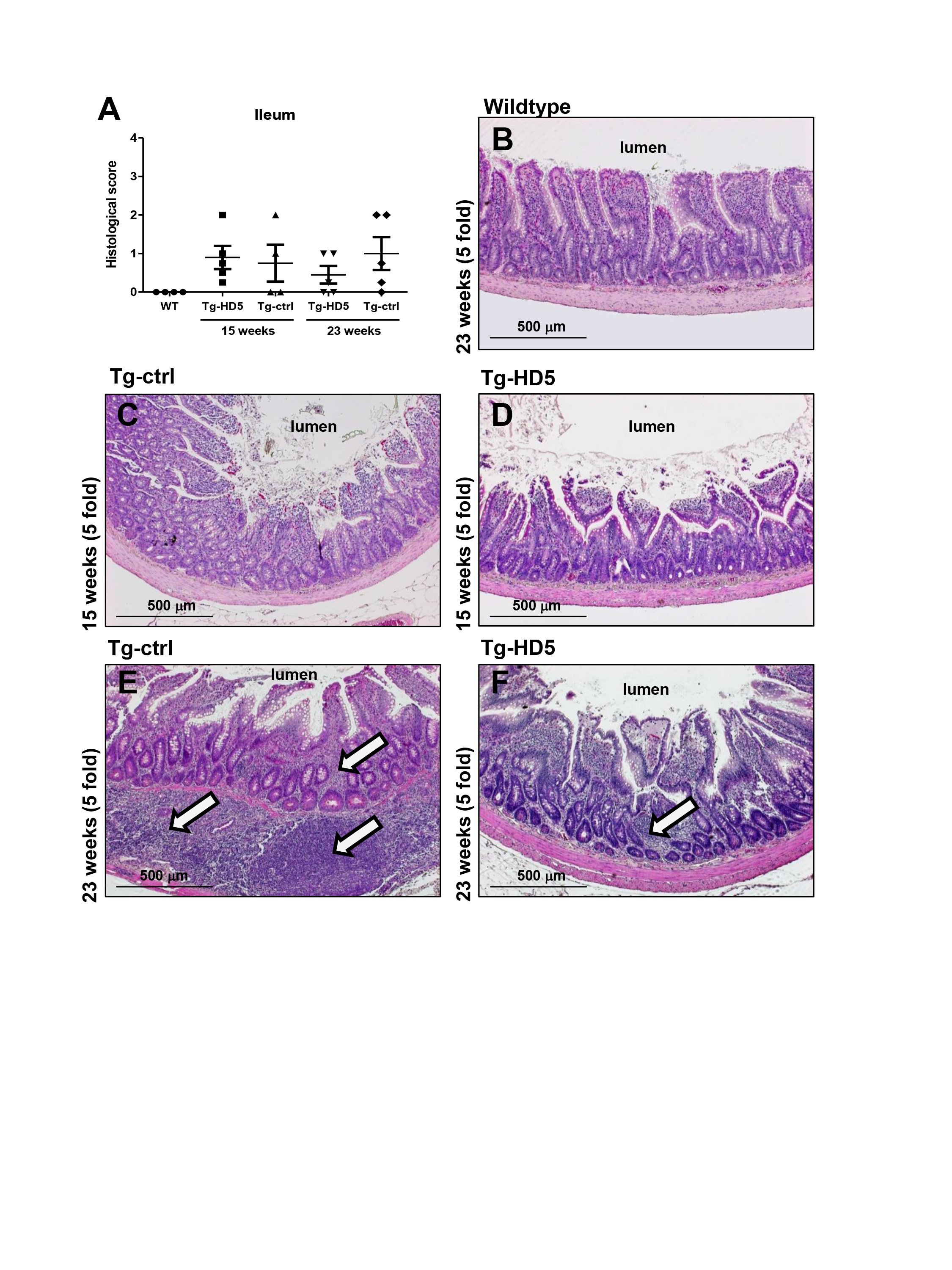

Supplement: S7 Fig — (A) Histological score of ileum. Representative images of WT-littermates 23 weeks (B), Tg-ctrl 15 weeks (C), Tg-HD5 15 weeks (D), Tg-ctrl, 23 weeks (E) and Tg-HD5 23 weeks (F). (A-F) No differences were observed between animal groups. Images representative for 5 rats each. White arrows indicate areas of a Peyer’s patch and lymphocyte influx. Original magnification 5-fold. Values are expressed as mean±SEM. (TIF) [file pone.0130811.s007.tif]

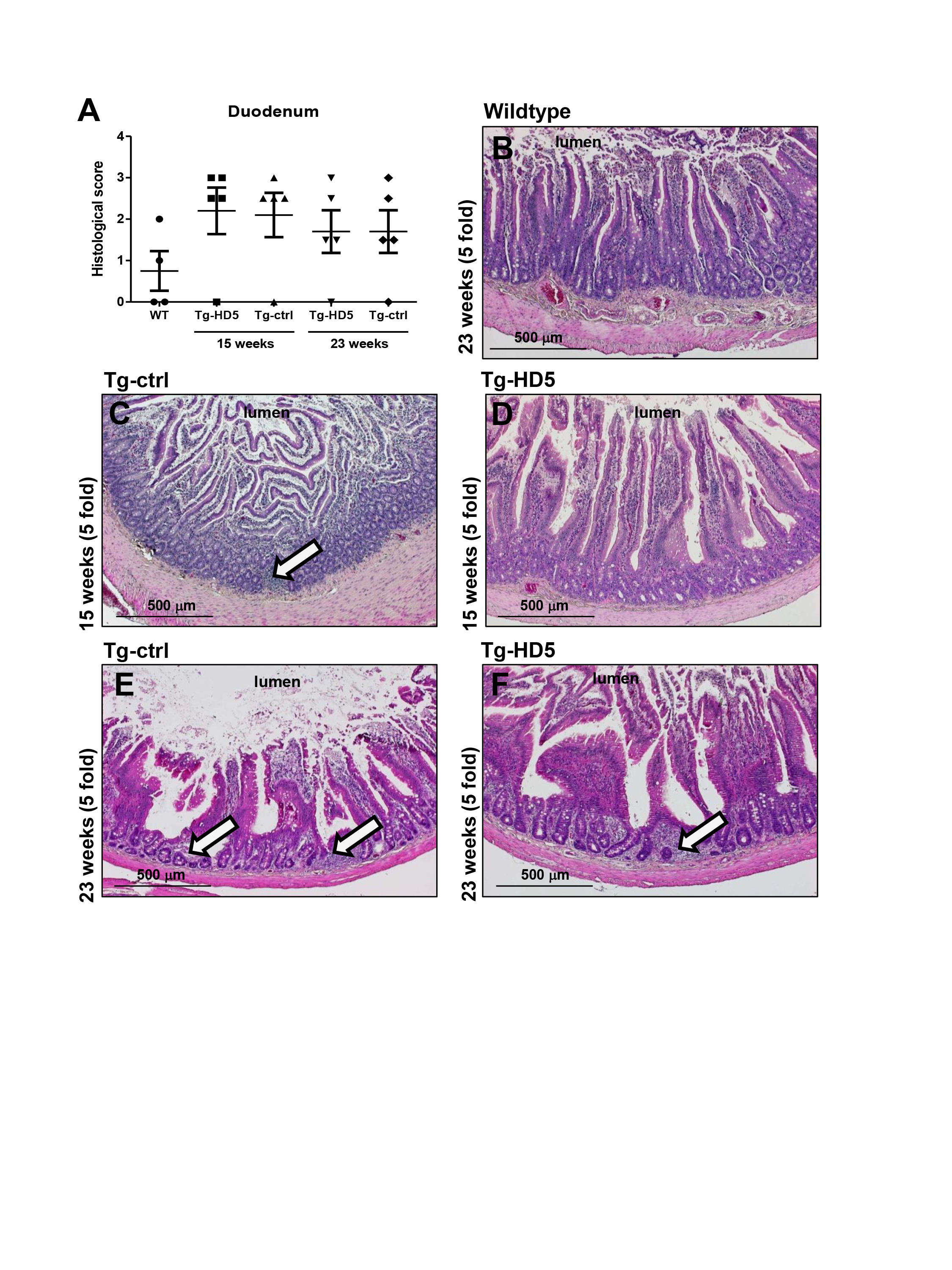

Supplement: S8 Fig — (A) Histological score of duodenum. Representative images of WT-littermates 23 weeks (B), Tg-ctrl 15 weeks (C), Tg-HD5 15 weeks (D), Tg-ctrl, 23 weeks (E) and Tg-HD5 23 weeks (F). (A-F) No differences were observed between animal groups. Images representative for 5 rats each. White arrows indicate areas of lymphocyte influx. Original magnification 5-fold. Values are expressed as mean±SEM. (TIF) [file pone.0130811.s008.tif]
